# Optimization of flavanonols heterologous biosynthesis in *Streptomyces albidoflavus*, and generation of auronols

**DOI:** 10.3389/fmicb.2024.1378235

**Published:** 2024-03-28

**Authors:** Patricia Magadán-Corpas, Suhui Ye, Annett Braune, Claudio J. Villar, Felipe Lombó

**Affiliations:** ^1^Research Group BIONUC (Biotechnology of Nutraceuticals and Bioactive Compounds), Departamento de Biología Funcional, Área de Microbiología, Universidad de Oviedo, Oviedo, Spain; ^2^IUOPA (Instituto Universitario de Oncología del Principado de Asturias), Oviedo, Spain; ^3^ISPA (Instituto de Investigación Sanitaria del Principado de Asturias), Oviedo, Spain; ^4^Research Group Intestinal Microbiology, German Institute of Human Nutrition Potsdam-Rehbruecke, Nuthetal, Germany

**Keywords:** aromadendrin, taxifolin, maesopsin, alphitonin, co-culture, flavanone 3-hydroxylase

## Abstract

Aromadendrin and taxifolin are two flavanonols (derived from the precursor naringenin) displaying diverse beneficial properties for humans. The carbon skeleton of these flavonoids may be transformed by the human gastrointestinal microbiota into other compounds, like auronols, which exert different and interesting biological activities. While research in flavonoids has become a certainly extensive field, studies about auronols are still scarce. In this work, different versions of the key plant enzyme for flavanonols biosynthesis, The flavanone 3-hydroxylase (F3H), has been screened for selecting the best one for the *de novo* production of these compounds in the bacterial factory *Streptomyces albidoflavus* UO-FLAV-004-NAR, a naringenin overproducer strain. This screening has rendered 2.6 μg/L of aromadendrin and 2.1 mg/L of taxifolin final production titers. Finally, the expression of the chalcone isomerase (CHI) from the gut bacterium *Eubacterium ramulus* has rendered a direct conversion (after feeding experiments) of 38.1% of (+)-aromadendrin into maesopsin and 74.6% of (+)-taxifolin into alphitonin. Moreover, *de novo* heterologous biosynthesis of 1.9 mg/L of alphitonin was accomplished by means of a co-culture strategy of a taxifolin producer *S. albidoflavus* and a CHI-expressing *Escherichia coli,* after the observation of the high instability of alphitonin in the culture medium. This study addresses the significance of culture time optimization and selection of appropriate enzymes depending on the desired final product. To our knowledge, this is the first time that alphitonin *de novo* production has been accomplished.

## 1 Introduction

Flavonoids are a family of polyphenolic compounds derived from plants (Kumar and Pandey, 2013; Das et al., 2021). These secondary metabolites are essential for the morphology and physiology of plants, displaying a wide range of functions, like cell growth regulation, pollinator insect attraction or protection against both biotic and abiotic stresses (Dias et al., 2021). Humans can benefit from flavonoid intake as these compounds show diverse bioactive properties, such as antioxidant (Chen et al., 2023; Liu et al., 2023), anti-inflammatory (Chen et al., 2023; Liu et al., 2023), antitumor (Redondo-Blanco et al., 2017) and cardioprotective (Espírito-Santo et al., 2023), among many others.

The chemical structure of flavonoids is composed of 15 carbon atoms (C6-C3-C6) forming two benzene rings (rings A and B) connected by a heterocyclic pyran ring (ring C) (Maleki et al., 2019; Marín et al., 2021). Flavonoids are grouped into different subclasses depending on the connection of rings B and C, the final conformation and oxidative status of ring C, and the total hydroxylation pattern. This structural variability of flavonoids is responsible for their diverse pharmacological and therapeutic potential (Maleki et al., 2019).

Flavonoids are present in small quantities in plants and accumulate only under certain environmental conditions. Consequently, their extraction constitutes a laborious and time-consuming process. On the other hand, their chemical synthesis is expensive and involves the use of toxic chemicals (Marín et al., 2021). For this reason, the heterologous production of flavonoids by microorganisms is becoming a valuable strategy as a fast and economic way of producing these compounds. In this context, the genus *Streptomyces* has been proven as a promising host for the enhanced production of flavonoids, given its ability to express multiple secondary metabolite biosynthetic gene clusters (Park et al., 2009, 2010, 2011; Marín et al., 2017, 2018, 2021; Hwang et al., 2021).

**Figure 1:**
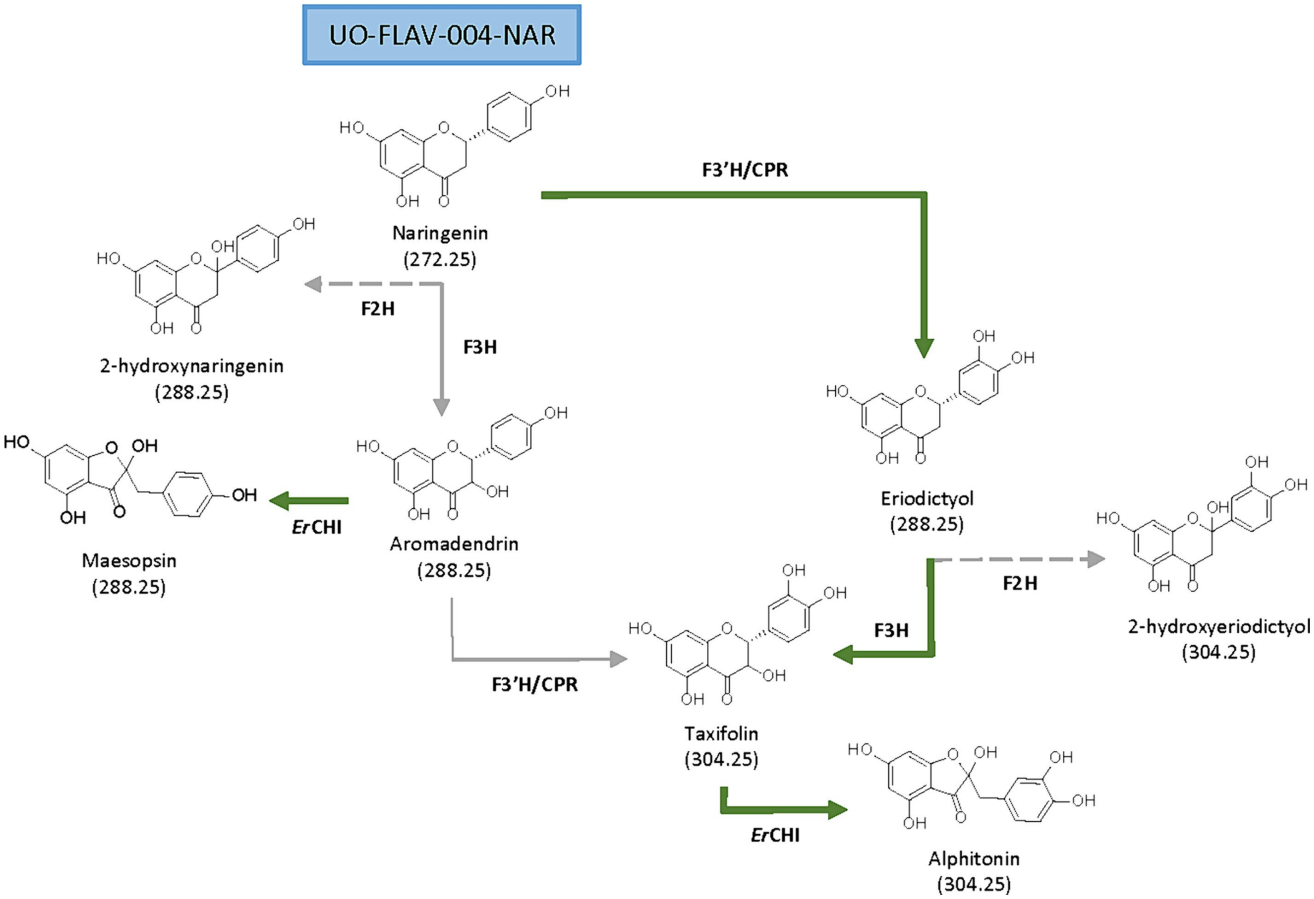
Proposed biosynthetic pathways for *de novo* production of flavanonols and auronols from naringenin. Main (green solid arrows) and secondary biosynthetic pathways (gray solid arrows), as well as side reactions (gray dashed arrows) are shown.

Aromadendrin (Figure 1), or dihydrokaempferol, is a flavanonol that exerts anti-inflammatory, antioxidant, and anti-diabetic properties (Lee et al., 2013). It has been reported to attenuate, *in vivo*, induced hepatic injury and hepatic fibrosis (Huang et al., 2023), and it shows a protective effect against experimental cardiac hypertrophy (Cui et al., 2018).

Taxifolin (Figure 1), also known as dihydroquercetin, is a flavanonol that, as aromadendrin, contains two stereocenters giving rise to four stereoisomers (Bernatova and Liskova, 2021; Das et al., 2021). Many pharmacological properties are attributed to taxifolin, such as anti-inflammatory, antioxidant (Liu et al., 2023), anti-Alzheimer, hepatoprotective, antiangiogenic, antimicrobial (Bernatova and Liskova, 2021; Das et al., 2021), and also cosmetic ones (Chen et al., 2023). Taxifolin has a higher antioxidant potential compared to other flavonoids, based on the disposition of its phenol groups and the elevated number of hydroxyl groups in its structure (Das et al., 2021).

Aurone derivates, auronols (Figure 1), are very interesting compounds due to their remarkable therapeutic potential as antiviral, immunosuppressive and chemopreventive agents, and also as inhibitors of multidrug resistance mechanisms (Elsinghorst et al., 2011). These auronols are flavonoid derivatives originating from a carbon skeleton reorganization in ring C, where this moiety is converted to a 5-carbon heterocyclic ring (Figure 1). In particular, alphitonin revealed significant antidiabetic and antioxidant properties (Kim et al., 2017), even more efficient than quercetin (Amić and Mastiľák Cagardová, 2023); and a maesopsin glycoside, maesopsin 4-O-β-glucoside, exhibited antitumor activity, both *in vitro* and *in vivo*, by inhibiting the growth of cancer cells (Pozzesi et al., 2011; Thuy et al., 2016). However, although auronols constitute a very promising group of compounds, yet few studies have been carried out on their therapeutic potential and their heterologous production.

The flavanone naringenin constitutes a central metabolite in flavonoid biosynthesis, being the common precursor for the biosynthesis of all the abovementioned compounds. From naringenin, the additional required genes constitute the main rate-limiting steps for both aromadendrin and taxifolin heterologous production (Lv et al., 2019; Gao et al., 2020; Yu et al., 2022). A flavanone 3-hydroxylase (F3H) is needed to convert naringenin into aromadendrin. This F3H, belonging to the 2-oxoglutarate-dependent dioxygenase family, exerts a low catalytic efficiency (Gao et al., 2020) and shows a 2-hydroxylase side activity (Marín et al., 2021). Subsequent conversion of aromadendrin into taxifolin is catalyzed by a flavonoid 3′-hydroxylase (F3’H). This is a membrane-bound cytochrome P_450_ monooxygenase that requires a cytochrome P_450_ reductase (CPR) for electron transfer (Leonard et al., 2006; Marín et al., 2017; Wu et al., 2022). In a previous work from our research group, the low activity of F3’H, due to its cytoplasmic insolubility and the need for reducing power, was addressed in *Streptomyces albidoflavus* J1074 by constructing a chimeric enzyme of F3’H and a reductase (Magadán-Corpas et al., 2023).

Flavonoids may be transformed by bacteria in the human gastrointestinal tract as part of the bacterial metabolism. In this way, aromadendrin and taxifolin are transformed into their respective auronol derivatives by the action of a chalcone isomerase from *Eubacterium ramulus* (*Er*CHI) (Braune et al., 2001, 2016). This reaction is a coenzyme-independent isomerization taking place in the presence or absence of oxygen (Braune et al., 2001). The *Er*CHI has been proven as enantioselective toward (+)-aromadendrin and (+)-taxifolin, catalyzing its C-ring contraction to maesopsin or alphitonin, respectively, by a ring opening-recyclation mechanism, with the corresponding chalcone and its di-keto tautomer as reaction intermediates (Elsinghorst et al., 2011; Braune et al., 2016).

Regarding this, it has been described that the presence of the biosynthetic precursor eriodictyol reduces the *Er*CHI-catalyzed isomerization of (+)-taxifolin into alphitonin to 51%, due to a competition for binding to the *Er*CHI active site. Also, the (+)-aromadendrin conversion into maesopsin is reduced to 84% in the presence of naringenin. This inhibition is reported to occur through a blockage of the *Er*CHI active site, with no further transformation of neither naringenin nor eriodictyol (Braune et al., 2016).

In this study, the biosynthesis of aromadendrin, taxifolin, maesopsin and alphitonin has been addressed in a naringenin overproducer *S. albidoflavus* J1074 strain. In this context, the auronols instability in the culture conditions has been identified as a major drawback for their heterologous production in *S. albidoflavus* and has been overcome by means of feeding with precursors for both maesopsin and alphitonin, and the co-culture of a taxifolin-producer *S. albidoflavus* strain with a CHI-expressing *E. coli* strain, after optimal F3H selection, for *de novo* alphitonin production.

## 2 Materials and methods

### 2.1 Genes, enzymes, antibiotics, and primers

Restriction enzymes and T4 DNA ligase were purchased from Thermo Fisher Scientific. Herculase II Fusion DNA polymerase was purchased from Agilent Technologies. NEBuilder^®^ HiFi DNA Assembly Master Mix for Gibson assembly was purchased from Thermo Fisher Scientific. The antibiotics used were ampicillin (100 μg/mL) (Sigma Aldrich; Madrid, Spain); apramycin (50 μg/mL for *S. albidoflavus* or 100 μg/mL for *E. coli*) (Thermo Fisher Scientific; MA, United States); nalidixic acid (50 μg/mL) (Acros Organics; Belgium) and thiostrepton (50 μg/mL) (Cayman Chemical; MI, United States).

Primers were synthesized by IDT Technologies (Leuven, Belgium). Sequences of primers used in this work are listed in Supplementary Table S1.

This work is based on the F3H from *Petroselinum crispum* (Genbank accession no. AY230248, and OR820614 for the optimized gene), the F3H from *Malus domestica* (Genbank accession no. AAX89397, and OR820613 for the optimized gene), the F3H from *Camellia sinensis* (Genbank accession no. AY641730, and OR820612for the optimized gene), and the CHI from *Eubacterium ramulus* (GenBank accession no. AIS36173, and OR820615 for the optimized gene). The encoding gene sequences were synthesized by Explora Biotech (Venice, Italy) after codon optimization for *Streptomyces* codon usage and removal of unique restriction sites for SEVA platform and Golden Standard cloning, as Golden Standard level 0 plasmids.

### 2.2 Plasmid construction

All the plasmids developed in this study have been constructed based in the plasmid library for *Streptomyces* developed in our group (Magadán-Corpas et al., 2023), following the Golden Standard Assembly (Blázquez et al., 2023) and listed in Supplementary Table S2. The details on the construction of each plasmid in this work are included in the Supplementary data file (Plasmid Construction section, page 4). All plasmids described were verified by restriction enzyme digestions and sequencing.

### 2.3 Strain generation

Exogenous DNA was introduced into *S. albidoflavus* by either protoplast transformation or intergeneric conjugation (Kieser et al., 2000). Strains containing integrative plasmids were selected based on their resistance to the corresponding antibiotics and/or by PCR.

The strain *S. albidoflavus* UO-FLAV-004-NAR carries the naringenin biosynthetic gene cluster (BGC) integrated into its ՓC31 chromosomal site (Pérez-Valero et al., 2023). Plasmids pSEVAUO-M22106-PcF3H, pSEVAUO-M22106-MdF3H and pSEVAUO-M22106-CsF3H, pSEVAUO-M21503-PcF3H-F3’H/CPR, pSEVAUO-M21503-CsF3H-F3’H/CPR and pSEVAUO-M21603-PcF3H-F3’H/CPR-ErCHI [Supplementary data file (Plasmid Construction section, page 4)] were independently integrated into the ՓBT1 integration site of the *S. albidoflavus* UO-FLAV-004-NAR strain, yielding *S. albidoflavus* UO-FLAV-004-PcARO, *S. albidoflavus* UO-FLAV-004-MdARO, *S. albidoflavus* UO-FLAV-004-CsARO, *S. albidoflavus* UO-FLAV-004-PcTAX, *S. albidoflavus* UO-FLAV-004-CsTAX and *S. albidoflavus* UO-FLAV-004-ALPH strains, respectively.

Plasmid pSEVAUO-M21302-ErCHI [Supplementary data file (Plasmid Construction section, page 4)] was integrated into the ՓBT1 integration site of *S. albidoflavus* UO-FLAV-004 strain, the same improved strain used in previous experiments, but lacking naringenin BGC (Pérez-Valero et al., 2023), yielding the strain *S. albidoflavus* UO-FLAV-004-ErCHI.

### 2.4 Bacterial strains and culture conditions

All strains used in this study are listed in Supplementary Table S2. *E. coli* strains were grown as previously specified (Magadán-Corpas et al., 2023). *S. albidoflavus* was grown at 30°C in yeast extract-malt extract (YEME) supplemented with 17% (w/v) sucrose for the preparation of protoplasts, and MA medium was used for conjugation (Fernández et al., 1998). *S. albidoflavus* was sporulated in Bennett medium (Kieser et al., 2000) supplemented with the corresponding antibiotics when necessary.

For flavonoid production, *S. albidoflavus* spores were quantified, and 10^7^ spores/ml were inoculated in shake flasks with 25 mL of NL333 pH 7.2 medium (Myronovskyi et al., 2014) and incubated at 30°C and 250 revolutions per minute (rpm) for 5 days, unless otherwise specified. Feedings with precursors were performed after 24 h of culture at a final concentration of 100 μM, and samples were collected after 5 days of total culture time (unless otherwise specified). Cultures were performed in triplicate.

Regarding the co-cultures of *S. albidoflavus* and *E. coli*, 10^7^ spores/mL of *S. albidoflavus* UO-FLAV-004-CsTAX strain were cultivated in NL333 pH 7.2 medium for 48 h at 30°C and 250 rpm. An inoculum of 350 μL from an *E. coli* JM109 pTI2 overnight culture was added to 35 mL of TSB with ampicillin and cultivated at 37°C and 250 rpm until OD reached 0.6. Then, IPTG 1 mM was added, and the culture was grown for another 4 h at 37°C and 250 rpm. Following this, 10 mL of the *E. coli* culture were centrifuged, the cell pellet resuspended in 1 mL of *S. albidoflavus* UO-FLAV-004-CsTAX culture aliquot and added to the *S. albidoflavus* flask. Samples were taken from the *S. albidoflavus* UO-FLAV-004-CsTAX culture immediately before adding *E. coli* as a control, and immediately after placing the co-culture. This process was carried out in triplicate.

### 2.5 Reagents and biochemicals

All solvents used for solid-phase extraction, High-Performance Liquid Chromatography with Diode Array Detection (HPLC-DAD) and High Performance Liquid Chromatography-High-Resolution Electrospray Ionization Mass Spectrometry (HPLC-HRESIMS) analysis were LC–MS grade from either Sigma-Aldrich or VWR Chemicals. Authentic standard compounds for identification and quantification by HPLC-HRESIMS and HPLC-DAD analyses were provided by Extrasynthese (Genay, France).

### 2.6 Flavonoid extraction

Spores from the different *S. albidoflavus* strains were incubated as previously described. Samples of 1 mL were taken from flasks. Flavonoids were recovered by an organic extraction with acetone (cellular pellet) and ethyl acetate (culture supernatant) as previously described (Magadán-Corpas et al., 2023). The final dry extract was reconstituted in 100 μL DMSO/MeOH (1:1, v/v), and the samples were centrifuged prior to HPLC injection.

For alphitonin and maesopsin experiments, sample extraction was performed immediately after their collection, with fresh solutions of organic solvents (acetone and ethyl acetate) acidified (Braune et al., 2016) with 0.1% formic acid.

### 2.7 HPLC analysis

Flavonoid identification was performed using either HPLC-DAD or HPLC-HRESIMS. The HPLC-DAD separation was conducted in a 1,260 Infinity (Agilent Technologies) HPLC system equipped with an analytical Pursuit XRs C18 column (50 × 4.0 mm, 5 μm, Agilent Technologies). Samples were run by an isocratic elution of 90% de-ionized water and 10% MeCN, both acidified with 0.1% formic acid, during 5.44 min, followed by a linear gradient from 10 to 35% of MeCN until min. 21.77, then maintained until min. 27.21. Next, a linear gradient from 35 to 100% MeCN was performed up to min. 43.54, followed by an isocratic elution until min. 55. Afterwards, a linear gradient from 100 to 10% MeCN was applied up to min. 56, and prolonged until the end of the program (min. 61).

The identification and quantification of the compounds by HPLC–HRESIMS and tandem mass spectrometry (MS/MS) was performed in an Ultra Performance Liquid Chromatography (UPLC) system (Dionex Ultimate 3000, Thermo Scientific, Madrid, Spain) equipped with an analytical RP-18 HPLC column (50 × 2.1 mm, Zorbax^®^ Eclipse Plus, 1.8 μm, Agilent Technologies, Madrid, Spain) as previously described (Marín et al., 2021). The obtained base peak chromatograms (BPCs) were extracted for the deprotonated ions of a set of flavonoids (271.0611 [M-H]^−^ (calculated for C_15_H_12_O_5_) corresponding to naringenin; 287.0561 [M-H]^−^ (calculated for C_15_H_12_O_6_) for eriodictyol, aromadendrin, 2-hydroxynaringenin and maesopsin; 303.0510 [M-H]^−^ (calculated for C_15_H_12_O_7_), corresponding to taxifolin and alphitonin) with a mass error range of 0.005 milli mass units (mmu) and the obtained extracted ion chromatograms (EICs) were compared with authentic commercial standards.

Compounds were quantified by comparing the peak area with that of a known amount of an authentic compound through a calibration curve. The production titers are given in μg/L or mg/L, and the mean value was calculated from three biological replicates.

### 2.8 Flavonoids stability tests

For alphitonin, 10 mL NL333 pH 7.2 in a 100 mL flask was spiked with 50 μM of commercial alphitonin, and 1 mL samples were taken immediately after the feeding, 4 h and 24 h post-feeding. The samples were immediately extracted with 800 μL of ethyl acetate freshly acidified with 0.1% formic acid, and this extraction was repeated. The collected organic phases were dried in a vacuum centrifuge. The dry extract obtained was reconstituted in 100 μL DMSO/MeOH 1:1 (v/v), and the samples were centrifuged prior to HPLC-DAD injection.

For taxifolin stability tests with cells, 25 mL NL333 pH 7.2 in a 250 mL flask were inoculated with 107 UFC/mL of *S. albidoflavus* UO-FLAV-004 strain, incubated for 48 h at 250 rpm and 30°C, and then fed with 10 μM of commercial taxifolin. Samples of 1 mL were taken after the feeding every 24 h for 5 days and extracted afterwards. The dry extract obtained was reconstituted in 100 μL DMSO/MeOH 1:1 (v/v), and 5 μL were used for HPLC-HRESIMS analysis. For taxifolin stability tests without cells, identical culture conditions were used, but samples were taken every 24 h for 4 days and extracted afterwards.

### 2.9 Accession numbers

Sequence data have been deposited to the GenBank databases under accession numbers OR820614 for *Petroselinum crispum* F3H, OR820613 for *Malus domestica* F3H, OR820612 for *Camellia sinensis* F3H, OR820615 for *Eubacterium ramulus* CHI and OR820616 for the Golden Standard level 1 receptor plasmid pSEVAUO-M22106, assembled for this study as part of the Golden Standard library developed for its use in actinomycetes (Magadán-Corpas et al., 2023).

### 2.10 Statistical analysis

A two-way ANOVA was used to compare cultivation data between different strains. A Sidak correction was used. The alpha threshold (and confidence level) selected for the data were 0.05 (95% confidence level). The data were expressed as the mean value ± standard error of mean (S.E.M.). The graphic representation of all cultivation data was carried out using GRAPHPAD PRISM software (version 9, GraphPad Software, San Diego, CA, United States).

## 3 Results

### 3.1 Heterologous biosynthesis and optimization of aromadendrin

Aromadendrin is produced directly from naringenin by a F3H-catalyzed hydroxylation at the position 3 of ring C (Figure 1). In a preceding work from our research group, the *Petroselinum crispum* F3H enzyme (*Pc*F3H), previously named as N3DOX, was used in *S. albidoflavus* J1074 for the biosynthesis of kaempferol, myricetin and quercetin (Marín et al., 2018), sharing all of them aromadendrin as a pathway precursor. For this study, the same *Pc*F3H enzyme was used, however, the assembly strategy was approached differently, having each gene its own promoter, RBS and terminator [Supplementary data file (Plasmid Construction section)]. The strain *S. albidoflavus* UO-FLAV-004-NAR, a genetic engineered *S. albidoflavus* J1074 strain improved for naringenin production and able to reach 3.4 mg/L of this compound (Pérez-Valero et al., 2023), was used for further modifications.

The resulting aromadendrin producer strain, *S. albidoflavus* UO-FLAV-004-PcARO [see Material and Methods; Supplementary data file (Plasmid Construction section)], was cultivated, and its extracts were analyzed by HPLC-HRESIMS. A strain carrying the empty vector pSEVAUO-M22106, *S. albidoflavus* UO-FLAV-004-NAR-M22106, was used as negative control.

**Figure 2:**
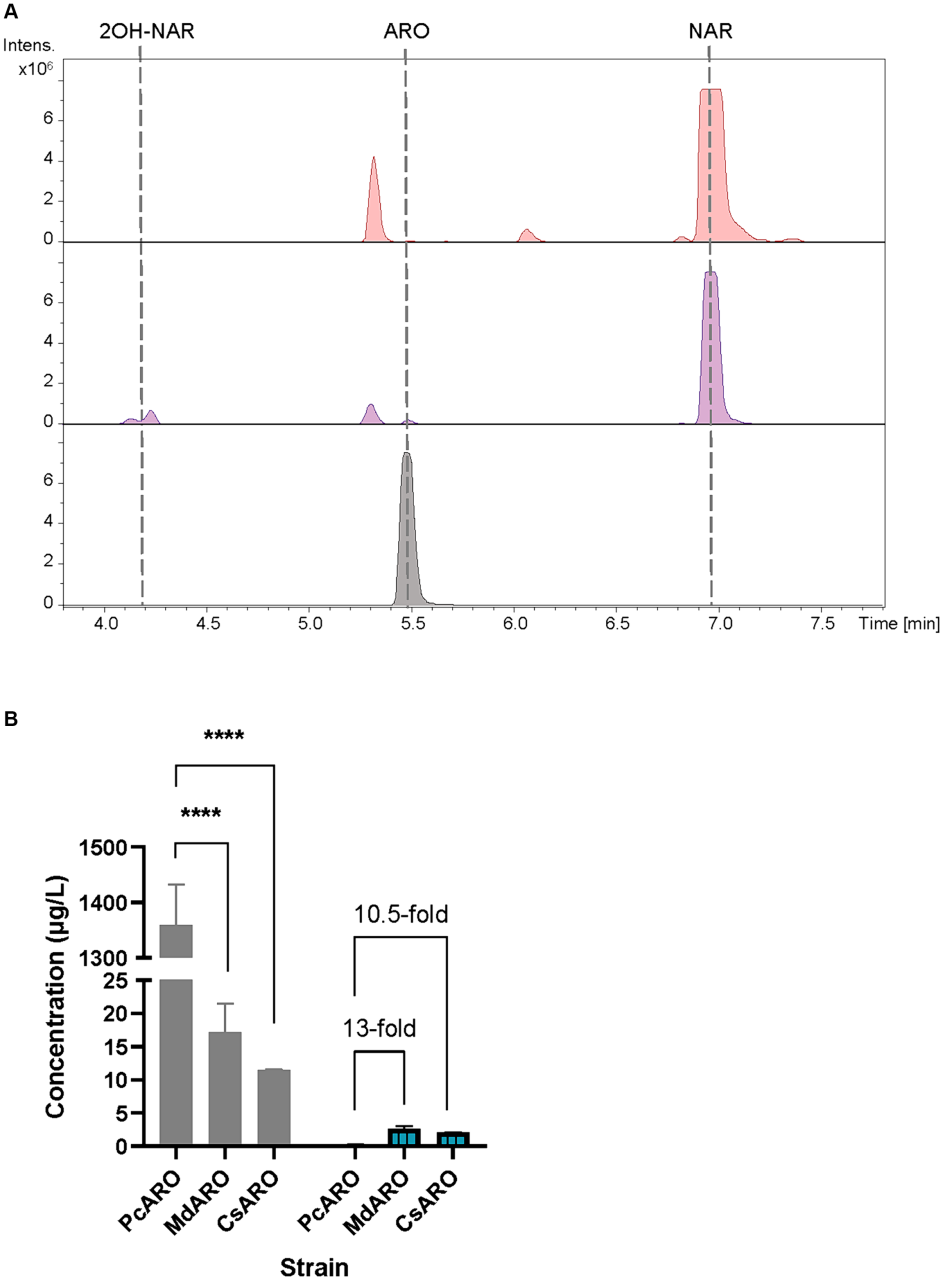
**(A)** HPLC-HRESIMS chromatograms of control strain *S. albidoflavus* UO-FLAV-004-NAR-M22106 (red) and the aromadendrin *de novo* producer strain *S. albidoflavus* UO-FLAV-004-PcARO (purple). Extracted ion chromatograms for naringenin, 2-hydroxynaringenin and aromadendrin. Aromadendrin commercial standard (black). **(B)** Compared *de novo* aromadendrin biosynthesis (blue/lines) and precursor naringenin accumulation (gray) after F3H screening of *S. albidoflavus* UO-FLAV-004-PcARO, UO-FLAV-004-MdARO, and UO-FLAV-004-CsARO strains. NAR, naringenin; ARO, aromadendrin; 2OH-NAR, 2-hydroxynaringenin. Asterisks indicate statistically significant differences (*p < 0.05; **p < 0.005; ***p < 0.0005; ****p < 0.0001).

The obtained base peak chromatograms (BPCs) were extracted for the aromadendrin mass peak. A differential peak [retention time (rt): 5.5 min] for the proposed m/z, that perfectly coelutes with the pure commercial standard of aromadendrin, was detected in the samples from the producer strain (Figure 2A). The quantification of aromadendrin production titers in the *S. albidoflavus* UO-FLAV-004-PcARO strain was 0.2 ± 0.09 μg/L. The BPCs were additionally extracted for the precursor naringenin mass peak (rt: 6.9 min). Albeit naringenin is partially transformed to aromadendrin, large quantities of naringenin remained in the extracts (Figure 2A), revealing the *Pc*F3H activity as the main bottleneck for aromadendrin production. The presence of the derivative product 2-hydroxynaringenin, generated due to the 2-hydroxylase (F2H) side activity of the F3H enzyme over naringenin, can also be detected in the chromatogram (rt: 4.2 min) (Figure 2A).

Therefore, two other F3Hs were selected from different plant species, *Malus domestica* (*Md*F3H) and *Camellia sinensis* (*Cs*F3H), and these were compared regarding their performance with *Pc*F3H. These two new constructions were generated using the same promoter than the previous one, as a way to achieve the same transcription levels for the three genes. Strains *S. albidoflavus* UO-FLAV-004-MdARO and *S. albidoflavus* UO-FLAV-004-CsARO [see Material and Methods; Supplementary data file (Plasmid Construction section)] were cultivated, and the extracts were compared for aromadendrin production together with the *S. albidoflavus* UO-FLAV-004-PcARO strain.

The obtained BPCs were extracted for aromadendrin and naringenin mass peaks. The recombinant strain expressing *Cs*F3H yielded 2.1 ± 0.03 μg/L of aromadendrin, similar to the titers reached with the *Md*F3H-expressing strain of 2.6 ± 0.65 μg/L (Figure 2B). This constitutes a remarkable 13-fold increase in aromadendrin production compared to the *Pc*F3H-containing strain (0.2 ± 0.09 μg/L) (Figure 2B). However, the precursor naringenin was still accumulated (Figure 2B) and the side product 2-hydroxynaringenin was detected (data not shown) in incubations with all strains, meaning that F3H remains as the main bottleneck for aromadendrin production.

### 3.2 Heterologous biosynthesis and optimization of taxifolin

Taxifolin can be obtained from aromadendrin through the action of the F3’H hydroxylase. This enzyme is responsible for the hydroxylation of aromadendrin B-ring at the 3′ position (Figure 1). F3’H from *Arabidopsis thaliana* was successfully tested in our group fused to a reductase from the same plant as a chimera (F3’H/CPR) for the enhanced production of eriodictyol from naringenin (Magadán-Corpas et al., 2023).

In parallel to the aromadendrin producer strain *S. albidoflavus* UO-FLAV-004-PcARO, the taxifolin producer strain *S. albidoflavus* UO-FLAV-004-PcTAX was also generated [see Material and Methods; Supplementary data file (Plasmid Construction section)]. To confirm the heterologous production of taxifolin, the strain *S. albidoflavus* UO-FLAV-004-PcTAX was cultivated, and extracts were analyzed by HPLC-HRESIMS. The strain *S. albidoflavus* UO-FLAV-004-NAR-M21503, carrying the empty vector, was used as the negative control.

**Figure 3:**
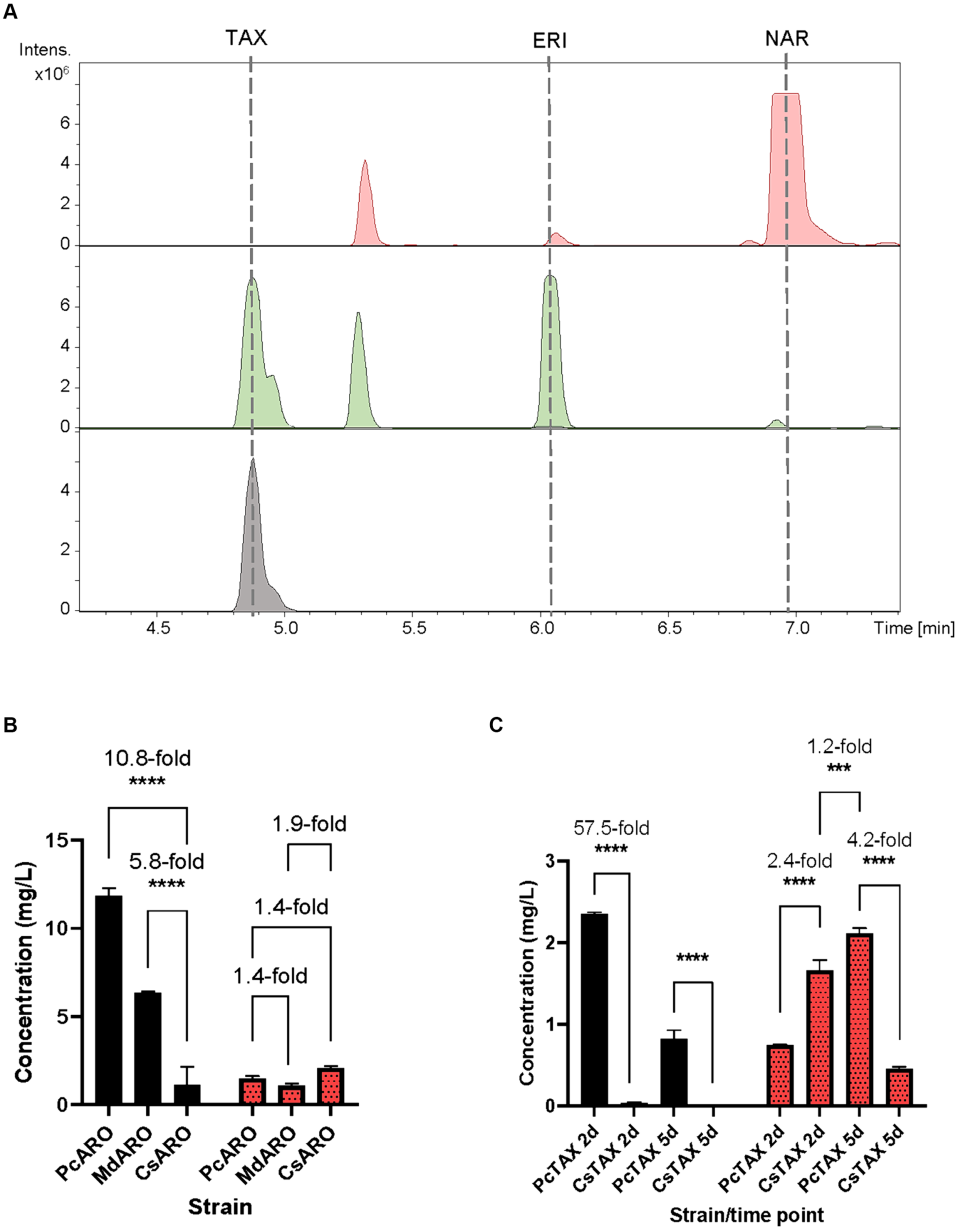
**(A)** HPLC-HRESIMS chromatograms of the control strain *S. albidoflavus* UO-FLAV-004-NAR-M21503 (red) and the taxifolin *de novo* producer strain *S. albidoflavus* UO-FLAV-004-PcTAX (green). Extracted ion chromatograms for naringenin, eriodictyol, aromadendrin and taxifolin. Taxifolin commercial standard (black). **(B)** Compared taxifolin biosynthesis (red/dots) and precursor eriodictyol accumulation (black) after feeding with 100 μM eriodictyol to *S. albidoflavus* UO-FLAV-004-PcARO, *S. albidoflavus* UO-FLAV-004-MdARO and *S. albidoflavus* UO-FLAV-004-CsARO. **(C)** Compared *de novo* taxifolin biosynthesis (red/dots) and precursor eriodictyol accumulation (black) of *S. albidoflavus* UO-FLAV-004-PcTAX and *S. albidoflavus* UO-FLAV-004-CsTAX. Samples taken 2 and 5 days after inoculation. The folds of the increased production are depicted. NAR, naringenin; TAX, taxifolin; ERI, eriodictyol. Asterisks indicate statistically significant differences (*p < 0.05; **p < 0.005; ***p < 0.0005; ****p < 0.0001).

The obtained BPCs were extracted for the naringenin (rt: 6.9 min), eriodictyol (rt: 6.1 min), aromadendrin (rt: 5.5 min) and taxifolin mass peaks. The obtained extracted ion chromatograms (EICs) revealed the presence of a peak (rt: 4.9 min) for the proposed m/z that was absent in the negative control *S. albidoflavus* UO-FLAV-004-NAR-M21503 strain, and that perfectly coeluted with the pure commercial standard of taxifolin (Figure 3A). Neither naringenin nor aromadendrin were detected in this strain. However, a peak corresponding to eriodictyol was detected resulting from the F3’H/CPR hydroxylation of naringenin (Figure 3A). The quantification of taxifolin production titers in the *S. albidoflavus* UO-FLAV-004-PcTAX led to 2.1 ± 0.11 mg/L. Considering the aromadendrin production titers of this study (0.2 ± 0.09 μg/L ≈ 7×10^−4^ μM), the amount of taxifolin achieved (2.1 ± 0.11 mg/L ≈ 6.9 μM) is much higher than the expected to be formed by direct conversion from aromadendrin. In addition to its established role in catalyzing the conversion of naringenin, F3H enzyme has been documented as capable of hydroxylating eriodictyol to produce the corresponding (2R/3R)-dihydroflavonol, taxifolin (Cheng et al., 2014). This finding, in conjunction with the prior observation of the substrate versatility of F3’H enzyme (converting naringenin to eriodictyol or aromadendrin into taxifolin) (Magadán-Corpas et al., 2023), has prompted us to propose an alternative pathway for the production of taxifolin from eriodictyol (Figure 1). The accumulation of the precursor eriodictyol in *S. albidoflavus* UO-FLAV-004-PcTAX, suggests that analogous to the situation observed in aromadendrin production, the primary limiting factor for taxifolin production, is associated with the enzymatic activity of the *Pc*F3H enzyme.

Henceforth, a feeding study involving the utilization of a 100 μM concentration of eriodictyol was conducted on three distinct strains, namely, *S. albidoflavus* UO-FLAV-004-MdARO, *S. albidoflavus* UO-FLAV-004-CsARO, and *S. albidoflavus* UO-FLAV-004-PcARO. This investigation aimed to assess the comparative capabilities of the respective F3H enzymes in these strains with regard to the synthesis of taxifolin following eriodictyol supplementation. Samples were analyzed by HPLC-DAD. The highest taxifolin titer was 2.1 ± 0.1 mg/L, yielded by *Cs*F3H, and constituting a 1.4-fold increased production compared to *Pc*F3H (1.5 ± 0.2 mg/L) and a 1.9-fold compared to *Md*F3H (1.1 ± 0.2 mg/L) (Figure 3B). A remarkable 10.8-fold decrease in the amount of eriodictyol that remained to be converted could be observed when the *Cs*F3H was compared with the *Pc*F3H, while a 5.8-fold decrease in the remained eriodictyol was detected when the comparison was made with the *Md*F3H (Figure 3B).

Subsequent to these results, a taxifolin-producer strain of *S. albidoflavus* was engineered, designated as *S. albidoflavus* UO-FLAV-004-CsTAX, in which the *Cs*F3H enzyme was integrated instead of the *Pc*F3H enzyme. A comparative evaluation was conducted between this newly created strain and the previously assayed *S. albidoflavus* UO-FLAV-004-PcTAX strain to assess their respective abilities in achieving enhanced taxifolin production. Samples were taken after 2 and 5 days of cultivation. Taxifolin and eriodictyol levels were measured by HPLC-HRESIMS. Divergent outcomes were noted contingent on the duration of the cultivation period. Specifically, after a 2-day cultivation period, strain *S. albidoflavus* UO-FLAV-004-CsTAX exhibited a taxifolin production that was 2.4-fold greater than that of strain *S. albidoflavus* UO-FLAV-004-PcTAX (1.7 ± 0.2 mg/L vs. 0.7 ± 0.005 mg/L) (Figure 3C), aligning with the results from the feeding experiments. Conversely, after 5 days of cultivation, strain *S. albidoflavus* UO-FLAV-004-PcTAX yielded a taxifolin production 4.2-fold higher than that of strain *S. albidoflavus* UO-FLAV-004-CsTAX (2.1 ± 0.1 mg/L vs. 0.5 ± 0.04 mg/L) (Figure 3C). Notably, the highest levels of residual eriodictyol were detected in the presence of the *Pc*F3H enzyme, amounting to 2.3 ± 0.04 mg/L and 0.8 ± 0.19 mg/L after 2 and 5 days of incubation, respectively. These levels displayed a decreasing trend over time, congruent with the observed trend of increasing taxifolin production. In contrast, minimal levels of eriodictyol were detected with the *Cs*F3H enzyme, registering at 0.04 ± 0.01 mg/L after 2 days of incubation, this indicating a 57.5-fold lower concentration of unconsumed precursor compared to *Pc*F3H. After 5 days, eriodictyol levels regarding the *Cs*F3H enzyme were below the detection limit (Figure 3C). These observations in eriodictyol consumption correlated with the earliest attainment of maximum taxifolin yield achieved by the *Cs*F3H enzyme. Following this rationale, it would be expected that taxifolin levels would remain relatively stable over time. However, a subsequent decrease in taxifolin levels was observed after 5 days of incubation in the case of the *Cs*F3H enzyme, which may be attributed to potential taxifolin degradation within the culture.

To substantiate this hypothesis, additional experiments were conducted wherein a 10 μM taxifolin supplement was added to the NL333 pH 7.2 culture medium, both in the presence and absence of *S. albidoflavus* UO-FLAV-004 cells [the same improved strain used in previous experiments, but lacking naringenin BGC (Pérez-Valero et al., 2023)]. The outcomes of these experiments demonstrated that taxifolin was indeed undergoing degradation or conversion by *S. albidoflavus* UO-FLAV-004. This assertion is supported by the conspicuous reduction in taxifolin levels, which was exclusively observed when the taxifolin supplementation occurred in the presence of the microbial cells (Supplementary Figure S1A), whereas taxifolin levels remained largely stable in the unseeded medium (Supplementary Figure S1B).

In summary, the optimal strategy for taxifolin production involved a 5-day incubation period of the *S. albidoflavus* UO-FLAV-004-PcTAX strain. However, it is important to note that this approach yielded only a modest 1.2-fold enhancement in taxifolin production compared to the highest production level achieved with *Cs*F3H enzyme, which occurred after a 2-day incubation period and exhibited substantially reduced precursor accumulation (Figure 3C).

### 3.3 Heterologous biosynthesis of alphitonin

A chalcone isomerase (CHI) is needed to catalyze the C-ring contraction of (+)-aromadendrin and (+)-taxifolin to the auronols maesopsin and alphitonin, respectively (Figure 1). In this study, the CHI from *Eubacterium ramulus* (*Er*CHI) was selected to carry out this conversion (Braune et al., 2016).

Following the development of the previous strains, the alphitonin producer *S. albidoflavus* UO-FLAV-004-ALPH strain was generated [see Material and Methods; Supplementary data file (Plasmid Construction section)]. To confirm the heterologous production of alphitonin, the strain *S. albidoflavus* UO-FLAV-004-ALPH was cultivated, its samples extracted with acidified organic solvents (see Materials and Methods) and analyzed by HPLC-HRESIMS. The strain *S. albidoflavus* UO-FLAV-004-PcTAX was used as the negative control.

**Figure 4:**
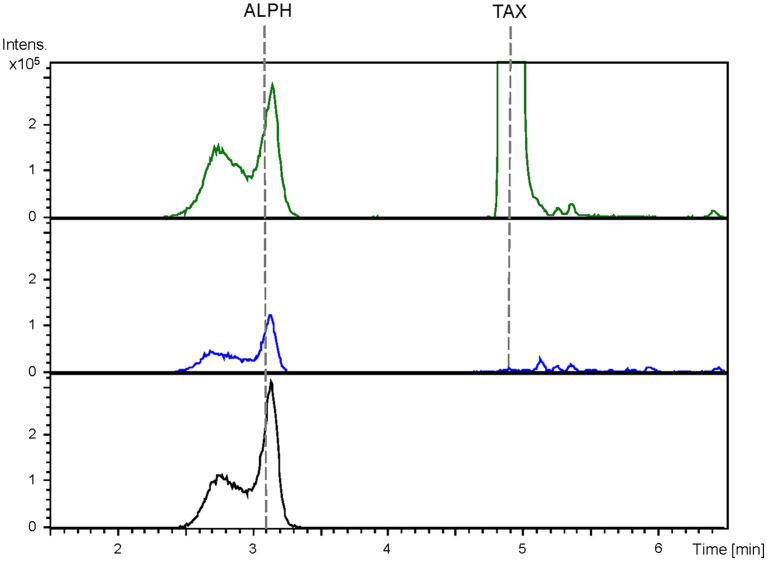
HPLC-HRESIMS chromatograms of control strain *S. albidoflavus* UO-FLAV-004-PcTAX (green) and the alphitonin *de novo* producer strain *S. albidoflavus* UO-FLAV-004-ALPH (blue). Extracted ion chromatograms for taxifolin and alphitonin. Alphitonin commercial standard (black). TAX, taxifolin; ALPH, alphitonin.

The obtained BPCs were extracted for the mass peaks corresponding to taxifolin and alphitonin. The obtained EICs unveiled the presence of a peak (rt: 3.1 min) corresponding to the proposed m/z value, which co-eluted with the pure commercial standard of alphitonin. However, it is noteworthy that the same peak was also identified in the negative control, the *S. albidoflavus* UO-FLAV-004-PcTAX strain (Figure 4). This observation suggests the absence of alphitonin production. Conversely, the peak corresponding to the precursor taxifolin (rt: 4.9 min) was conspicuously absent in the extracts obtained from the strain *S. albidoflavus* UO-FLAV-004-ALPH (Figure 4). Moreover, no additional new peaks were detected, indicating the consumption of taxifolin as a consequence of *Er*CHI heterologous expression in this strain.

To investigate this matter, an initial examination of alphitonin stability under our specified culture conditions was undertaken. In these experiments, fresh culture medium devoid of bacterial presence was supplemented with 50 μM of commercial alphitonin. Subsequently, it was subjected to incubation conditions identical to those employed in the production cultures, and the concentration of alphitonin was monitored at time intervals of 0, 4, and 24 h. The outcomes of this investigation confirmed the spontaneous degradation of alphitonin in culture medium, as evidenced by a progressive decline in alphitonin concentrations over time, ultimately culminating in its complete disappearance after 24 h of incubation (Supplementary Figure S2).

Subsequent stability assays aimed to mitigate alphitonin decomposition during culture were conducted. These efforts encompassed attempts to enhance alphitonin stability by altering the extraction conditions, specifically by employing higher concentrations of formic acid (0.1, 0.2, and 0.5%) in the organic solvents. Additionally, the supplementation of vitamin C (0.5 mM and 5 mM) was explored as a potential stabilizer in the NL333 medium (Zhang et al., 2022). Furthermore, investigations involved incubation of alphitonin in different media (R5A, TSB, and LB) and modifying the pH of NL333 medium from 7.2 to 6.8 (Zhang et al., 2022). Regrettably, none of these strategies yielded improved recoveries of alphitonin, as the results did not show any significant enhancement (data not shown).

### 3.4 Conversion of taxifolin and aromadendrin into alphitonin and maesopsin, respectively, by an *Er*CHI-expressing *Streptomyces albidoflavus* strain

Given the established instability of alphitonin under our culture conditions, an inquiry arose regarding whether the absence of discernible distinctions between the alphitonin-producing strain and the control strain, in relation to the alphitonin peak, could be attributed more to the alphitonin decomposition rather than its non-conversion. In pursuit of clarifying this matter, an experimental approach was employed. Specifically, a strain of *S. albidoflavus* carrying only the *Er*CHI transcription unit, designated as strain *S. albidoflavus* UO-FLAV-004-ErCHI, was utilized.

To elucidate the matter, strain *S. albidoflavus* UO-FLAV-004-ErCHI was employed in feeding cultures with a 100 μM concentration of taxifolin. As negative controls, cultures of the same strain were performed without taxifolin supplementation, and the strain *S. albidoflavus* UO-FLAV-004 was also cultured under both conditions. Samples were collected at designated time points of 0, 0.5, 1.5, 4, and 24 h post-feeding, promptly subjected to extraction, and subsequently analyzed using HPLC-DAD.

**Figure 5:**
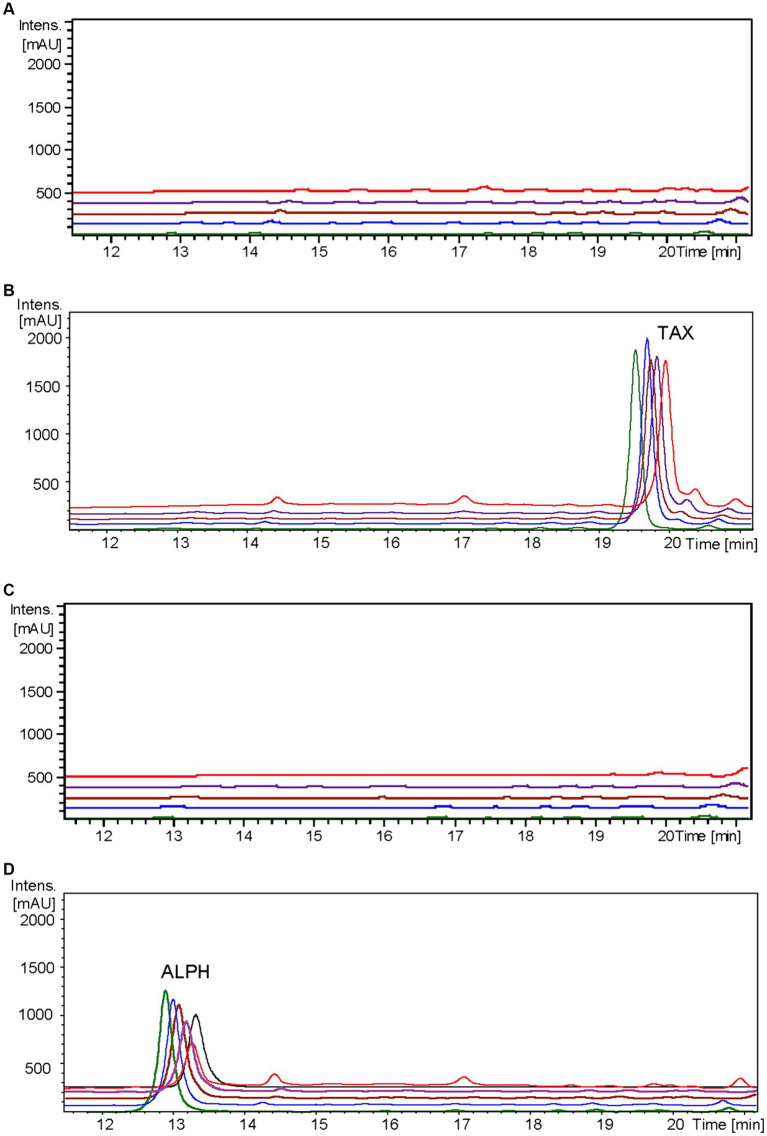
HPLC-DAD chromatograms at 279–281 nm of the time course for alphitonin biosynthesis after taxifolin feeding to *S. albidoflavus* UO-FLAV-004-ErCHI strain. **(A)** UO-FLAV-004 with DMSO, **(B)** UO-FLAV-004 with 100 μM taxifolin feeding, **(C)** UO-FLAV-004-ErCHI strain with DMSO, and **(D)** UO-FLAV-004-ErCHI with 100 μM taxifolin feeding. Samples are taken at 0 h (green), 0.5 h (blue), 1.5 h (brown), 4 h (purple), and 24 h (red) post-feeding. Alphitonin commercial standard (black). ALPH, alphitonin; TAX, taxifolin.

In the resulting UV chromatograms, a distinctive peak corresponding to alphitonin (rt: 13.1 min) was consistently identified subsequent to taxifolin feeding in all samples derived from the *S. albidoflavus* UO-FLAV-004-ErCHI strain (Figure 5). This alphitonin peak exhibited a maximal conversion rate of 74.6% from the total taxifolin provided at time 0 h, with declining concentrations at subsequent time points: 63.4, 59.5, 46.3, and 27% at 0.5, 1.5, 4, and 24 h, respectively. No remaining taxifolin was detected in any of the samples obtained from the strain *S. albidoflavus* UO-FLAV-004-ErCHI after the feeding. Conversely, in all samples overtime from the strain *S. albidoflavus* UO-FLAV-004, taxifolin (rt: 19.6 min) was found to accumulate, with no alphitonin production (Figure 5). These findings indicate the immediate conversion of taxifolin into alphitonin catalyzed by *Er*CHI, followed by rapid degradation of alphitonin within the culture.

Significantly, when samples from the control conditions were subjected to HPLC-HRESIMS analysis, the peak detected in the previous section of this study in both the control and the alphitonin-producing strains, coeluting with alphitonin standard, was conspicuously absent (Supplementary Figure S3). The potential identity of this peak will be discussed further in the subsequent sections.

**Figure 6:**
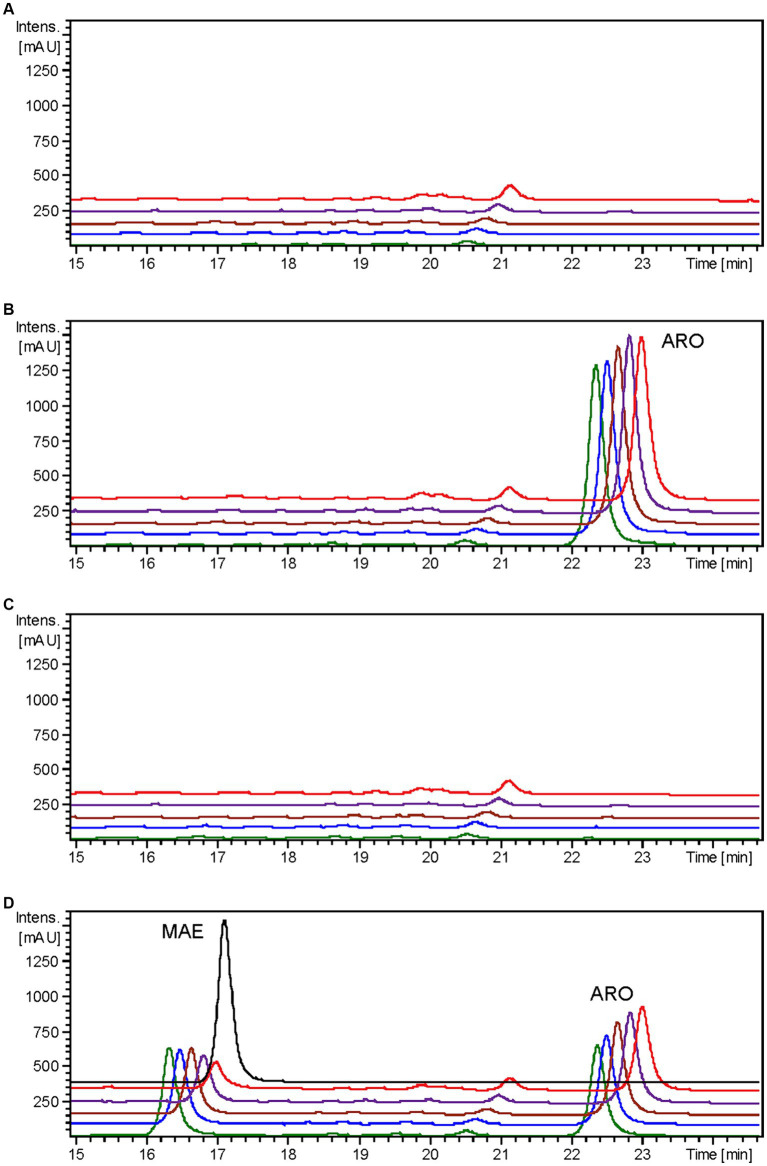
HPLC-DAD chromatograms at 279–281 nm of the time course for maesopsin biosynthesis after aromadendrin feeding to *S. albidoflavus* UO-FLAV-004-ErCHI strain. **(A)** UO-FLAV-004 with DMSO, **(B)** UO-FLAV-004 with 100 μM aromadendrin feeding, **(C)** UO-FLAV-004-ErCHI strain with DMSO, and **(D)** UO-FLAV-004-ErCHI with 100 μM aromadendrin feeding. Samples are taken at 0 h (green), 0.5 h (blue), 1.5 h (brown), 4 h (purple), and 24 h (red) post-feeding. Maesopsin commercial standard (black). MAE, maesopsin; ARO, aromadendrin.

It has been previously documented that *Er*CHI has the capacity to catalyze reactions involving (+)-aromadendrin, yielding the auronol maesopsin (Braune et al., 2016). Consequently, an analogous experiment was conducted, but with the addition of 100 μM of aromadendrin to the cultures. In this investigation, a distinct peak corresponding to maesopsin (rt: 16.4 min) was consistently identified following aromadendrin supplementation in all samples derived from the *S. albidoflavus* UO-FLAV-004-ErCHI strain (Figure 6). Maesopsin production reached a maximum conversion rate of 38.1% at time 0 h, with subsequent reductions in maesopsin concentrations observed over time: 32.2, 26.8, 19.3, and 11.7% at 0.5, 1.5, 4, and 24 h, respectively. However, in this particular instance, aromadendrin (rt: 22.4 min) was detected in all samples from the *S. albidoflavus* UO-FLAV-004-ErCHI strain following the feeding experiment (Figure 6). This represented 44, 43.7, 43, 42.6, and 41% of the unutilized precursor. Consequently, *Er*CHI expressed in *S. albidoflavus* demonstrated the capability to convert (+)-aromadendrin into maesopsin, albeit with a lesser degree of efficiency compared to the conversion of taxifolin into alphitonin.

### 3.5 *Streptomyces albidoflavus/Escherichia coli* co-cultures for heterologous *de novo* alphitonin biosynthesis

In summary of the previous experiments conducted in this study, it was observed that the *de novo* alphitonin generated by a single bacterial strain underwent degradation within the culture before becoming detectable. To overcome this issue, a single input of taxifolin is needed, as this taxifolin is promptly transformed into alphitonin, only to be subsequently degraded at a rapid pace. Consequently, in order to facilitate *de novo* alphitonin production, a co-culture system was established. This system involved a taxifolin-producing strain of *S. albidoflavus* in conjunction with *E. coli* JM109 pTI2 strain, the latter carrying the *Er*CHI gene (Braune et al., 2016).

The selection of the *S. albidoflavus* UO-FLAV-004-CsTAX strain for the co-culture was based on its demonstrated proficiency as an early-stage taxifolin producer (within 2 days) with minimal accumulation of the precursor eriodictyol. This choice was made considering that eriodictyol competes with taxifolin for binding to the active site of *Er*CHI enzyme. Both the *S. albidoflavus* UO-FLAV-004-CsTAX strain and the *E. coli* JM109 pTI2 strain were cultured separately and subsequently combined in a single flask, as detailed in the Materials and Methods section. Samples for production analysis were obtained from individual cultures as controls and immediately following the establishment of the co-culture, as the available taxifolin was promptly converted into alphitonin.

**Figure 7:**
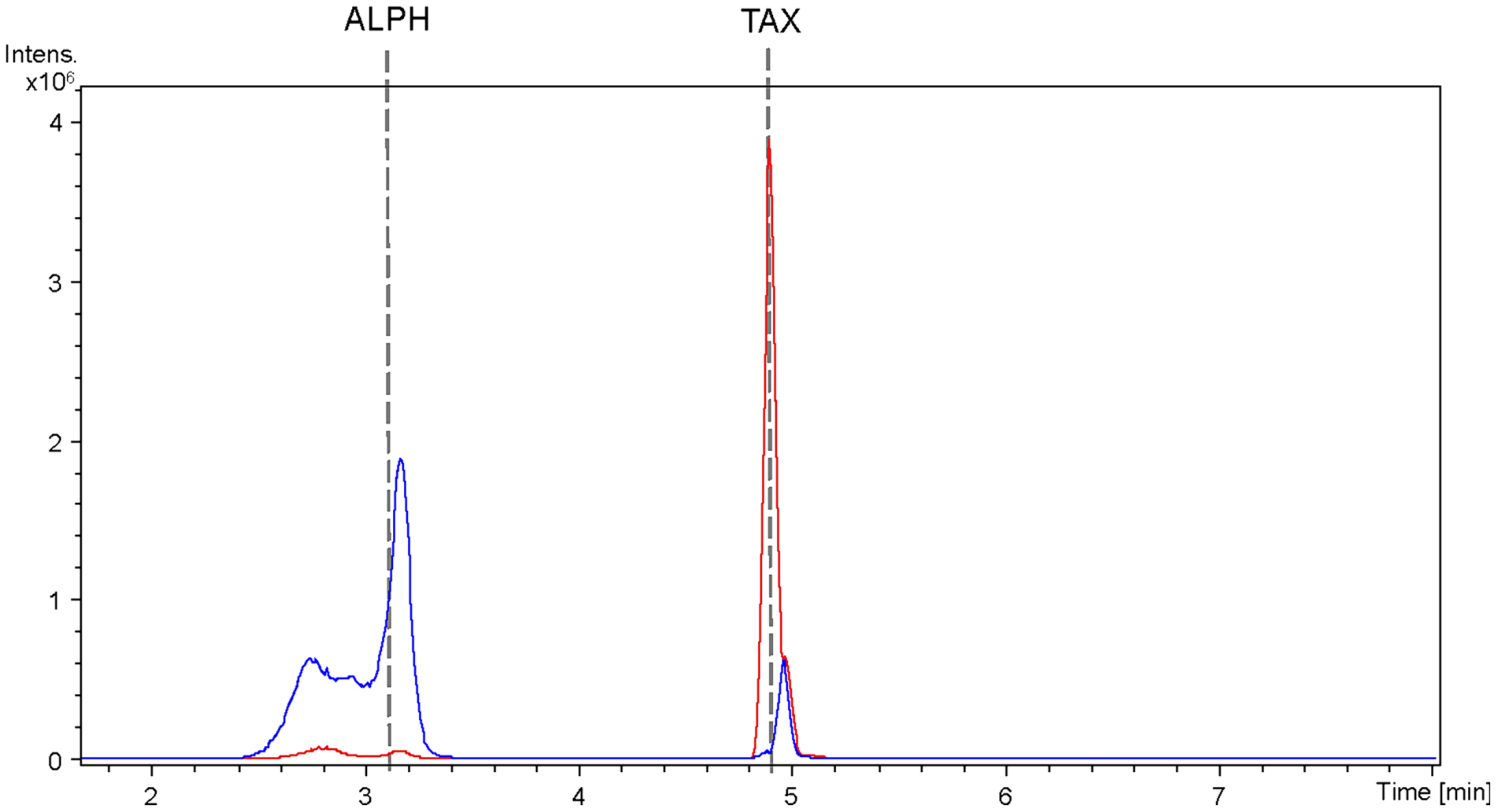
HPLC-HRESIMS extracted ion chromatograms of samples from the co-culture between the taxifolin producer strain *S. albidoflavus* UO-FLAV-004-CsF3H and the *E. coli Er*CHI-expressing strain JM109 pTI2, for the *de novo* alphitonin biosynthesis. Samples were taken from the *S. albidoflavus* culture before establishing the co-culture (red) and immediately after (blue) and chromatograms extracted for taxifolin and alphitonin m/z. TAX, taxifolin; ALPH: alphitonin.

The extracted samples were subjected to analysis using HPLC-HRESIMS. BPCs were extracted for the mass peak corresponding to taxifolin and alphitonin. In the case of the strain *S. albidoflavus* UO-FLAV-004-CsTAX cultivated as a single culture, the taxifolin concentration reached 1.4 ± 0.3 mg/L, while alphitonin production was not detected (Figure 7). However, upon the introduction of the *E. coli* strain into the culture and subsequent immediate sampling, taxifolin became undetectable, and alphitonin was identified at a concentration of 1.9 ± 0.7 mg/L (Figure 7). This outcome unequivocally confirmed the conversion of taxifolin into alphitonin.

## 4 Discussion

In previous investigations, the *de novo* biosynthesis of several flavonoids originating from naringenin in *S. albidoflavus* has been established (Marín et al., 2017, 2018, 2021; Pérez-Valero et al., 2023). In the present study, a genetically engineered *S. albidoflavus* J1074 strain, optimized for the production of 3.4 mg/L of naringenin (designated as *S. albidoflavus* UO-FLAV-004-NAR) (Pérez-Valero et al., 2023), was utilized, alongside a modular cloning platform. Consequently, this approach led to enhancements in the *de novo* synthesis of aromadendrin and taxifolin, as well as the production of two auronols, maesopsin and alphitonin, with the latter being produced *de novo* as well.

In this study, the reconstruction of the biosynthesis pathway for the flavanonols aromadendrin and taxifolin was undertaken in *Streptomyces albidoflavus* J1074 (Figure 1). During the course of this investigation, it was observed that both the enzymes, F3H and the chimeric enzyme F3’H/CPR, exhibited substrate flexibility. Consequently, it was demonstrated the capacity to produce taxifolin via two distinct enzymatic pathways, contingent upon the order of enzymatic reactions. In the primary pathway, the chimeric F3’H/CPR catalyzes the initial conversion of naringenin to eriodictyol, followed by the subsequent hydroxylation of eriodictyol by F3H to generate taxifolin. Conversely, in the secondary route, F3H catalyzes the initial conversion of naringenin to aromadendrin, which is then further converted into taxifolin by F3’H/CPR (Figure 1). This dual pathway arrangement allows for the maximization of taxifolin production.

The chimeric enzyme F3’H/CPR demonstrates high efficiency when acting upon both substrates, as evidenced by the absence of naringenin or aromadendrin accumulation in the cultures of the taxifolin-producing strain (Figure 3A). Nonetheless, our findings establish that the enzyme F3H serves as the primary limiting factor in the production of aromadendrin and taxifolin. This conclusion is affirmed by the substantial accumulation of precursor compounds, namely naringenin and eriodictyol, detected in the strains engineered for aromadendrin and taxifolin production, respectively (Figures 2A, 3A).

In an effort to enhance the production of aromadendrin and taxifolin, two additional F3Hs sourced from *Malus domestica* (*Md*F3H) and *Camellia sinensis* (*Cs*F3H) were assessed for their potential to exhibit increased activity and reduced 2-hydroxylase side activity. In the context of aromadendrin production, both *Cs*F3H and *Md*F3H demonstrated a similarly enhanced performance when acting upon naringenin, with *Md*F3H outperforming *Pc*F3H by yielding a 13-fold greater quantity of aromadendrin (Figure 2B).

Notwithstanding the improvements achieved in aromadendrin production, F3H still emerged as the limiting factor, as evidenced by the accumulation of naringenin and 2-hydroxynaringenin. Therefore, further efforts would be needed to optimize F3H functionality. It has been previously documented in the literature that F3H activity has evolved from flavonol synthase (FLS) in plants, potentially through a transitional phase involving bifunctional FLS/F3H intermediate, concurrently with the existence of bifunctional FLS/F2H intermediate (Li et al., 2020). Studies have reported on site-directed mutagenesis efforts targeting key amino acids within canonical FLS, F3H, and bifunctional enzymes, leading to shifts from F3H activity to either F2H or FLS activities (Li et al., 2020; Fu et al., 2023). Numerous other approaches have been explored, involving site-directed mutagenesis that resulted in alterations in catalytic efficiency, substrate affinity, or regio/stereoselectivity (Choi et al., 2015; Zhou et al., 2022; Sui et al., 2023; Xu et al., 2023).

In this context, it is advisable to conduct structural studies at the protein level focusing on F3H, with the aim of identifying critical amino acid residues that can be modified to enhance F3H activity while concurrently diminishing its F2H side activity. Such modifications would hold promise for future applications in the biosynthesis of flavonoids.

In the context of taxifolin production, the strain *S. albidoflavus* UO-FLAV-004-CsTAX, carrying the *Cs*F3H enzyme, generated 1.7 ± 0.2 mg/L of taxifolin. This quantity represented a 2.4-fold increase in taxifolin production compared to its counterpart harboring the *Pc*F3H enzyme, and this difference was observed after a cultivation period of 2 days (Figure 3C). Conversely, the strain *S. albidoflavus* UO-FLAV-004-PcTAX yielded 2.1 ± 0.1 mg/L of taxifolin after an additional 3 days of cultivation, marking the highest recorded production of taxifolin *de novo* in the course of this study (Figure 3C). Therefore, while there was no enhancement in taxifolin production, the screening of F3H enzymes proved valuable in reducing the cultivation time and precursor accumulation, while maintaining comparable final levels of taxifolin production.

Given the observed trends of taxifolin degradation subsequent to the complete consumption of precursors, coupled with the achievement of maximum taxifolin yield in strain *S. albidoflavus* UO-FLAV-004-CsTAX (Figure 3C), and the challenges encountered in *de novo* alphitonin production using a single strain (Figure 4), an investigation into the stability of these target compounds within the culture environment was conducted. Experiments involving the addition of taxifolin to both seeded and unseeded cultures revealed that, unlike auronols, taxifolin exhibited stability under the culture conditions, however, it was susceptible to conversion or degradation by *S. albidoflavus* cells (Supplementary Figure S1). Nevertheless, this issue was successfully mitigated by optimizing the cultivation duration, which varied depending on the specific F3H variant employed (Figure 3C). In contrast, auronols exhibited pronounced instability within cell-free culture conditions (Supplementary Figure S2).

Confirmation of the conversion of aromadendrin into maesopsin and taxifolin into alphitonin by *Er*CHI was obtained through feeding experiments conducted on a strain of *S. albidoflavus* exclusively expressing the *Er*CHI enzyme. These experiments provided evidence of the immediate conversion of both precursor compounds into their respective auronols, subsequently followed by the rapid degradation of these auronols (Figures 5, 6). Furthermore, the results from these experiments indicated that a *S. albidoflavus* strain expressing *Er*CHI displayed greater efficiency in converting taxifolin into alphitonin compared to aromadendrin conversion into maesopsin. Specifically, when equal amounts (100 μM) of the respective substrate were introduced into the culture, *Er*CHI achieved a 74.6% conversion of taxifolin into alphitonin, whereas only 38.1% of aromadendrin was transformed into maesopsin. This finding aligns with earlier findings involving cell-free extracts of *Er*CHI expressed in *E. coli*, which demonstrated 60% activity with aromadendrin in comparison to taxifolin (Braune et al., 2016). *Er*CHI exhibited its highest activity within very short and early time frames, with maximum activity observed at the time of substrate feeding and no further activity subsequently recorded. This was evidenced by the complete conversion of taxifolin immediately after feeding (Figure 5) and the consistent presence of essentially the same quantity of unconverted aromadendrin throughout the corresponding experiment (Figure 6).

As all the attempts to stabilize alphitonin through modifications in culture and extraction conditions proved unsuccessful, a co-culture strategy was implemented between the strain *S. albidoflavus* UO-FLAV-004-CsTAX and an *E. coli* strain expressing the *Er*CHI (Braune et al., 2016). This approach ultimately yielded successful results, achieving *de novo* production of alphitonin at a concentration of 1.9 ± 0.7 mg/L, with no accumulation of its precursor, (+)-taxifolin (Figure 7). Optimization in the selection of the F3H enzyme and culture duration ensured a substantial taxifolin supply with negligible precursor accumulation (57.5-fold lower eriodictyol accumulation compared to its counterpart *Pc*F3H) (Figure 3C). Consequently, the absence of the precursor prevented inhibition of *Er*CHI during the catalysis of taxifolin conversion into alphitonin.

For maesopsin, the limited titers of aromadendrin generated by the respective producer strains, coupled with precursor accumulation resulting from diminished F3H activity, will require further improvement at the enzyme level prior to embarking on the *de novo* production attempts.

Finally, it is pertinent to acknowledge and provide a plausible rationale for the conspicuous peak observed in Figure 4 and again in Figure 7, which co-elutes with alphitonin standard in the control taxifolin producer strain. Considering the previously reported F2H side activity of F3H (Marín et al., 2021), a product generated from naringenin associated with this activity was detected in the aromadendrin producer strain, leading to the production of the derivative 2-hydroxynaringenin (Figures 1, 2A). Notably, the 2-hydroxynaringenin peak was absent in samples from the control *S. albidoflavus* UO-FLAV-004-NAR-M22106 strain (Figure 2A), which lacks F3H. Its identity was further confirmed through comparison with a pure commercial standard of 2-hydroxynaringenin. Intriguingly, both 2-hydroxynaringenin and maesopsin standards exhibited co-elution in both HPLC-HRESIMS and HPLC-DAD analyses, and they shared identical m/z, MS/MS, and UV/Vis spectra (Supplementary Figure S4).

In light of these findings, it is proposed that in the taxifolin producer strain, the F2H side activity associated with F3H may also act upon eriodictyol, leading to the formation of a corresponding 2-hydroxylated intermediate compound, 2-hydroxyeriodictyol (Figure 1). This side intermediate compound would co-elute with alphitonin, mirroring the co-elution of 2-hydroxynaringenin with maesopsin. Unfortunately, a 2-hydroxyeriodictyol standard is not commercially available. However, the observation that these peaks, corresponding to 2-hydroxynaringenin and 2-hydroxyeriodictyol, exclusively appear in chromatograms when F3H and the corresponding precursor (naringenin and/or eriodictyol) are present strongly supports this hypothesis. Consequently, this unequivocally associates this activity with the F3H enzyme (Supplementary Figure S5).

In this study, we have comprehensively addressed the significance of culture time optimization and enzyme selection based on the desired final product. Effective optimization strategies were employed to achieve complete consumption of precursors during the production of taxifolin and alphitonin. Nevertheless, additional optimization methodologies need to be explored to maximize the production of aromadendrin and maesopsin, particularly in light of the limited efficiency exhibited by F3H and *Er*CHI enzymes for their respective substrates. Moreover, this study represents the first report of *de novo* heterologous biosynthesis of alphitonin accomplished through the judicious selection of optimal F3H enzyme and the establishment of a co-culture between *S. albidoflavus* J1074 and *E. coli*, owing to the observed high instability of this compound in cell-free culture.

## Data availability statement

The datasets presented in this study can be found in online repositories. The names of the repository/repositories and accession number(s) can be found in the article/Supplementary material.

## Author contributions

PM-C: Investigation, Writing – original draft. SY: Supervision, Writing – review & editing. AB: Writing – review & editing. CV: Supervision, Writing – review & editing. FL: Funding acquisition, Supervision, Writing – review & editing.
