# Supplementary material for: Optimization of flavanonols heterologous biosynthesis in Streptomyces albidoflavus, and generation of auronols
Source: Front Microbiol. 2024 Mar 28;15:1378235. doi: 10.3389/fmicb.2024.1378235 (PMC11007074; doi:10.3389/fmicb.2024.1378235)
Supplement: Supplementary file 1 [file Data_Sheet_1.pdf]

Supplementary Material

1 Supplementary Tables

Supplementary Table 1. Primers used in this study.

Primer	Sequence 5'-3'
UNS5 fw	GAGCCAACTCCCTTTACAACCTCACTC
UNS1 rev	GAGACGAGACGAGACAGCCTGAGAATG
Cargo UNS5 rev	CTCTAACGGACTTGAGTGAGGTTGTAAAGGGAGTTGG CTCGGGACCCTCTGAACAAATCCAGATG
pBBR1 UNS3 fw	GCACTGAAGGTCCTCAATCGCACTGGAAACATCAAG GTCGCTACCGGCGCGGCAGCGTT
UNS2 fw	GCTGGGAGTTCGTAGACG
UNS3 rev	CGACCTTGATGTTTCCAG
CRISPR F1 UNS1 fw bis	CATTACTCGCATCCATTCTCAGGCTGTCTCGTCTCGTC TCCTTTTCCGCTGCATAACCC
CRISPR F1 UNS2 rev	GCTTGGATTCTGCGTTTGTTTCCGTCTACGAACTCCCA GCTCATGGCTCTGCCCTCGG

Supplementary Table 2. Plasmids and strains used in this work (Ap^R: ampicillin-resistance; Tsr^R: thioestrepton-resistance; Am^R: apramycin-resistance).

Plasmid	Use in this work	Reference or source
pCRBluntAm-Tsr	Source of Am-Tsr^R	(Magadán-Corpas et al., 2023)
pSEVA23g19g1	Source of pBBR1 and 1AI2 gadget	(Blázquez et al., 2023)
pSEVAUO-21003	Source of ϕBT1 integrase	(Magadán-Corpas et al., 2023)
pSEVAUO-C41012	Source of oriT-traJ	(Magadán-Corpas et al., 2023)
pSEVAUO-M22106	Golden Standard level 1 receptor vector (pBBR1-ϕBT1-Am/Tsr^R-1AI2) for cargo cloning	This work (OR820616)
pSEVA181SP25	Source of SP25 (Level 0 Golden Standard)	(Magadán-Corpas et al., 2023)
pSEVA181SP43	Source of SP43 (Level 0 Golden Standard)	(Magadán-Corpas et al., 2023)
pSEVA181RiboJ-RBS	Source of RiboJ-RBS (Level 0 Golden Standard)	(Magadán-Corpas et al., 2023)
pIDTSMARTttsbib	Source of ttsbib (Level 0 Golden Standard)	(Magadán-Corpas et al., 2023)
pSEVA181PcF3H	Source of *Pc*F3H (Level 0 Golden Standard)	EXPLORA
pSEVA181MdF3H	Source of *Md*F3H (Level 0 Golden Standard)	EXPLORA
pSEVA181CsF3H	Source of *Cs*F3H (Level 0 Golden Standard)	EXPLORA
pSEVAUO-M22106-PcF3H	pBBR1-ϕBT1-Am-Tsr^R. Level 1 Golden Standard plasmid containing [SP25]-*Pc*F3H	This work
pSEVAUO-M22106-MdF3H	ϕBT1, pBBR1, Am-Tsr^R. Level 1 Golden Standard plasmid containing [SP25]-*Md*F3H	This work
pSEVAUO-M22106-CsF3H	ϕBT1, pBBR1, Am-Tsr^R. Level 1 Golden Standard plasmid containing [SP25]-*Cs*F3H	This work
pSEVAUO-M21206-F3′H-CPR	ϕBT1, pUC, Am-Tsr^R. Level 1 Golden Standard plasmid containing [SF14]-F3'H/CPR	(Magadán-Corpas et al., 2023)
pSEVAUO-M21503	Golden Standard level 2 receptor vector (pUC-ϕBT1-Ap-Tsr^R-A13B) for cargo cloning	(Magadán-Corpas et al., 2023)
pSEVAUO-M21503-PcF3H-F3'H/CPR	pUC- ϕBT1-Ap-Tsr^R. Level 2 Golden Standard plasmid containing [SP25]-*Pc*F3H-[SF14]-F3'H/CPR	This work
pSEVAUO-M21503-CsF3H-F3'H/CPR	pUC- ϕBT1-Ap-Tsr^R. Level 2 Golden Standard plasmid containing [SP25]-*Cs*F3H-[SF14]-F3'H/CPR	This work
pSEVAUO-M21302	Golden Standard level 1 receptor vector (pUC-ϕBT1-Am^R-3AI4) for cargo cloning	(Magadán-Corpas et al., 2023)
pSEVA181ErCHI	Source of *Er*CHI (Level 0 Golden Standard)	EXPLORA
pSEVAUO-M21302-ErCHI	pUC- ϕBT1-Am^R-3AI4. Level 1 Golden Standard plasmid containing [SP43]-*Er*CHI	This work
pSEVAUO-M21603	Golden Standard level 2 receptor vector (pUC-ϕBT1-Ap-Tsr^R-B14C) for cargo cloning	(Magadán-Corpas et al., 2023)
pSEVAUO-M21603-PcF3H-F3'H/CPR-ErCHI	pUC- ϕBT1-Ap-Tsr^R-B14C. Level 2 Golden Standard plasmid containing [SP25]-*Pc*F3H-[SF14]-F3'H/CPR-[SP43]-*Er*CHI	This work
**Strain**	**Use in this work**	**Reference or source**
Escherichia coli Top10	Routine sub-cloning and DNA propagation	Invitrogen

Escherichia coli ET12567/pUZ8002	Routine conjugation	Life Science
Escherichia coli JM109 pTI2	*E. coli* strain carrying *Er*CHI for the co-cultures	(Braune et al., 2016)
S. albidoflavus UO-FLAV-004	*S. albidoflavus* J1074 lacking the pseudo-attB site for the ϕC31 recombination system and with 3 BGCs deleted by CRISPR/Cas9	(Pérez-Valero et al., 2023)
S. albidoflavus UO-FLAV-004-M21302	*S. albidoflavus* UO-FLAV-004 with pSEVAUO-M21302 empty receptor vector integrated into ϕBT1 site	This work
S. albidoflavus UO-FLAV-004-ErCHI	*S. albidoflavus* UO-FLAV-004 with *Er*CHI integrated into ϕBT1 site	This work
S. albidoflavus UO-FLAV-004-NAR	*S. albidoflavus* UO-FLAV-004 with naringenin BGC integrated into ϕC31 site	(Pérez-Valero et al., 2023)
S. albidoflavus UO-FLAV-004-NAR-M22106	*S. albidoflavus* UO-FLAV-004-NAR with pSEVAUO-M22106 empty receptor vector integrated into ϕBT1 site	This work
S. albidoflavus UO-FLAV-004-NAR-M21503	*S. albidoflavus* UO-FLAV-004-NAR with pSEVAUO-M21503 empty receptor vector integrated into ϕBT1 site	This work
S. albidoflavus UO-FLAV-004-PcARO	*S. albidoflavus* UO-FLAV-004-NAR with *Pc*F3H integrated into ϕBT1 site	This work
S. albidoflavus UO-FLAV-004-MdARO	*S. albidoflavus* UO-FLAV-004-NAR with *Md*F3H integrated into ϕBT1 site	This work
S. albidoflavus UO-FLAV-004-CsARO	*S. albidoflavus* UO-FLAV-004-NAR with *Cs*F3H integrated into ϕBT1 site	This work
S. albidoflavus UO-FLAV-004-PcTAX	*S. albidoflavus* UO-FLAV-004-NAR with *Pc*F3H-F3'H/CPR integrated into ϕBT1 site	This work
S. albidoflavus UO-FLAV-004-CsTAX	*S. albidoflavus* UO-FLAV-004-NAR with *Cs*F3H-F3'H/CPR integrated into ϕBT1 site	This work
S. albidoflavus UO-FLAV-004-ALPH	*S. albidoflavus* UO-FLAV-004-NAR with *Pc*F3H-F3'H/CPR-*Er*CHI integrated into ϕBT1 site	This work

2 Supplementary Data

Plasmid construction

Construction of Golden Standard level 1 pSEVAUO-M22106 receptor vector

This plasmid was assembled as part of the plasmid library developed in our group for its use in *Streptomyces* (Magadán-Corpas et al., 2023). The Am-Tsr antibiotic resistance marker cassette, conferring resistance to apramycin and thiostrepton, was amplified from pCRBluntAm-Tsr (Magadán-Corpas et al., 2023) with primers "UNS5 fw" and "UNS1 rev". Gadget 1AI2 for Golden Standard assembly and *E. coli* pBBR1 origin of replication were amplified together from plasmid pSEVA23g19g1 (Magadán-Corpas et al., 2023) with primers "Cargo UNS5 rev" and "pBBR1 UNS3 fw". Integrase ϕBT1 was amplified from plasmid pSEVAUO-21003 (Magadán-Corpas et al., 2023) with primers "UNS2 fw" and "UNS3 rev". oriT-traJ for conjugation was amplified from plasmid pSEVAUO-C41012 (Magadán-Corpas et al., 2023) with primers "CRISPR F1 UNS1 fw bis" and "CRISPR F1 UNS2 rev". Finally, a 4 fragment Gibson assembly was performed to give rise to the level 1 receptor vector pSEVAUO-M22106. This sequence data has been submitted to the GenBank databases under accession number OR820616.

Construction of pSEVAUO-M22106-PcF3H, pSEVAUO-M22106-MdF3H and pSEVAUO-M22106-CsF3H

Enzymes *Pc*F3H, *Md*F3H and *Cs*F3H were codon optimized, prepared for Golden Standard assembly and ordered synthetic as level 0 plasmids. These genes were assembled as Golden Standard level 1 plasmids. Level 1 plasmids were generated from level 1 receptor plasmid pSEVAUO-M22106, level 0 plasmids pSEVA181SP25, pSEVA181RiboJ-RBS, pIDTSMARTttsbib (Magadán-Corpas et al., 2023) and the corresponding level 0 for each CDS (pSEVA181PcF3H, pSEVA181MdF3H or pSEVA181CsF3H).

Construction of pSEVAUO-M21503-PcF3H-F3'H/CPR and pSEVAUO-M21503-CsF3H-F3'H/CPR

Level 1 Golden Standard plasmids pSEVAUO-M22106-PcF3H and pSEVAUO-M22106-CsF3H were individually assembled together with the level 1 pSEVAUO-M21206-F3′H-CPR plasmid (Magadán-Corpas et al., 2023), into the pSEVAUO-M21503 level 2 receptor vector (Magadán-Corpas et al., 2023), yielding plasmids pSEVAUO-M21503-PcF3H-F3'H/CPR and pSEVAUO-M21503-CsF3H-F3'H/CPR.

Construction of pSEVAUO-M21603-PcF3H-F3'H/CPR-ErCHI

Enzyme *Er*CHI was codon optimized for Golden Standard assembly and ordered synthetic as a level 0 plasmid. This gene was assembled as a Golden Standard level 1 plasmid. Level 1 plasmid pSEVAUO-M21302-ErCHI was generated from level 1 receptor vector pSEVAUO-M21302 and level 0 plasmids pSEVA181SP43, pSEVA181RiboJ-RBS, pSEVA181ErCHI and pIDTSMARTttsbib. The level 2 pSEVAUO-M21603-PcF3H-F3'H/CPR-ErCHI plasmid was assembled in a single Golden Standard reaction from level 2 receptor plasmid pSEVAUO-M21603 (Magadán-Corpas et al., 2023) and level 1 plasmids pSEVAUO-M22106-PcF3H, pSEVAUO-M21206-F3′H-CPR and pSEVAUO-M21302-ErCHI.

Sequences of promoters, rbs-riboJ and terminator

Promoter SF14 (5’-3’):

CCTATCCAGGAGATATTATGAGTTACGTAGACCTACGCCTTGACCTTGATGAGGCGGCGTGAGCT
ACAATCAATACTCGATT

Promoter SP25 (5’-3’):

TGTTCACATTCGAACCGTCTCTGCTTTGACAGAGGTAGGCACGCTCATGTAAAGTCGTGGCC

Promoter SP43 (5’-3’):

TGTTCACATTCGAACCGTCTCTGCTTTGACACGGACAAGCGCTATGGTGTAAAGTCGTGGCC

riboJ (capital letters)-rbs SR41 (lowercase letters) (5’-3’):

AGCTGTCACCGGATGTGCTTTCCGGTCTGATGAGTCCGTGAGGACGAAACAGCCTCTACAAATAA
TTTTGTTTAAtctaagtaaggagtaggctg

Terminator ttsbib (5’-3’):

AAAAAAAAAGCGCCGCAACTGCGGCGCTTTTTTTTTT

3 Supplementary Figures

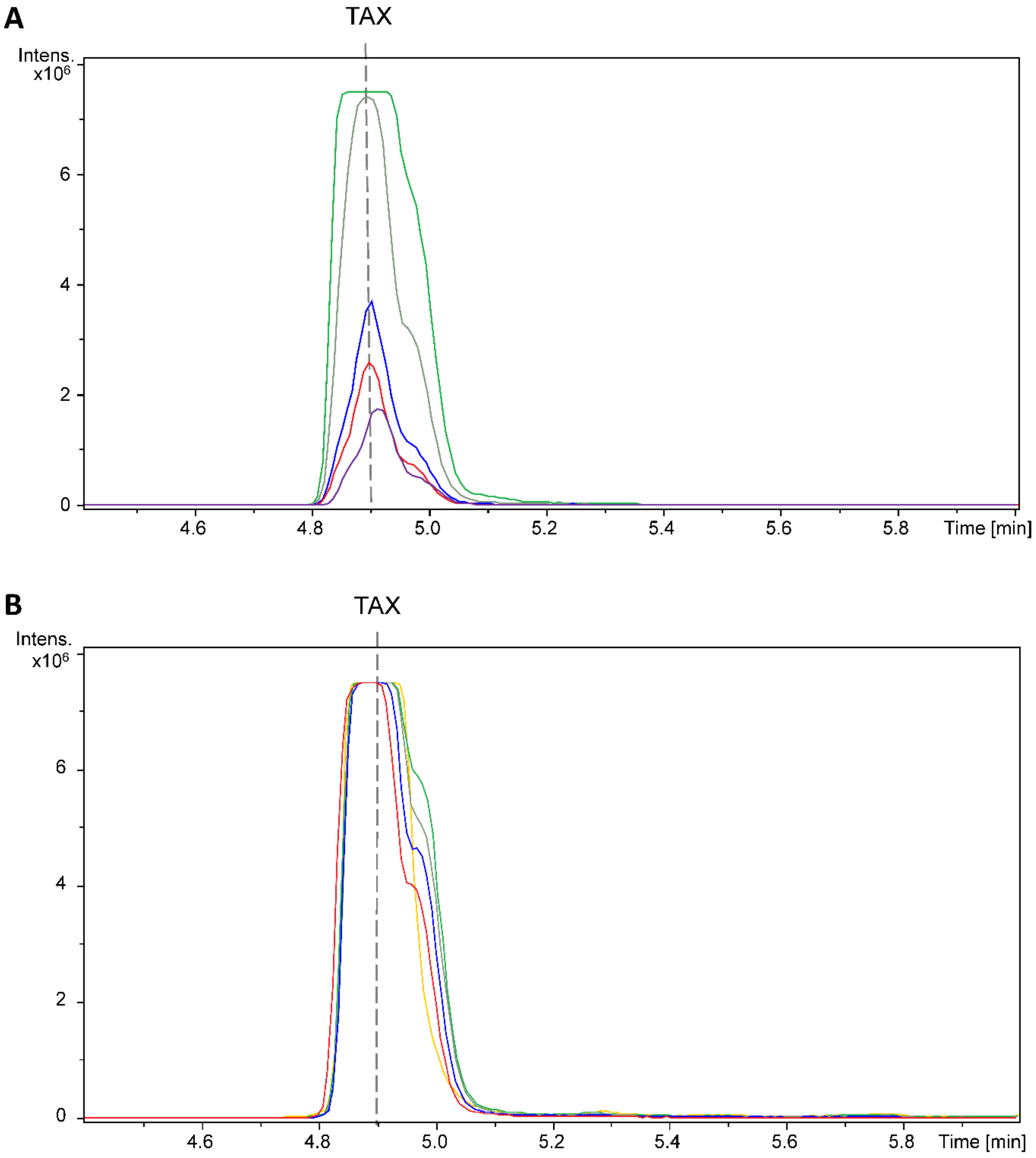

Supplementary Figure 1. HPLC-HRESIMS chromatograms extracted for taxifolin m/z, corresponding to taxifolin degradation experiments after 10 µM commercial taxifolin feeding to **(A)** NL333 pH 7.2 medium inoculated with 10^7 UFC/mL *S. albidoflavus* UO-FLAV-004 strain. Samples taken every 24h along 5 days: day 1 (green), day 2 (grey), day 3 (blue), day 4 (red) and day 5 (purple).

(B) NL333 pH 7.2 medium devoid of cells. Samples taken every 24h along 4 days: day 0 (yellow), day 1 (green), day 2 (grey), day 3 (blue) and day 4 (red). TAX, taxifolin.

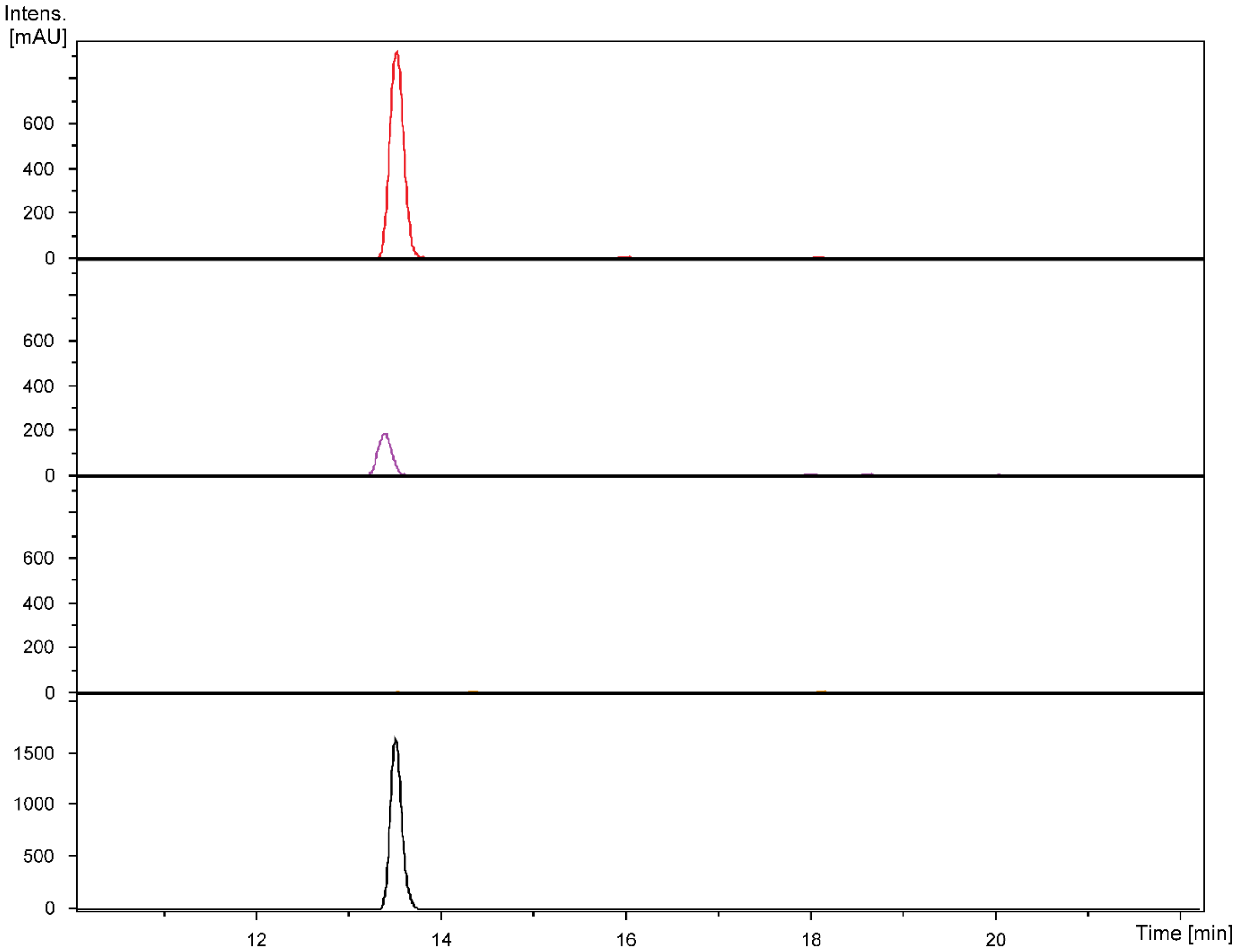

Supplementary Figure 2. HPLC-DAD chromatograms at 279-281 nm of a cell-free NL333 pH 7.2 culture medium spiked with 50 µM commercial alphitonin. Samples collected at times 0 h (red), 4 h (purple) and 24 h (yellow) after compound addition. Below, commercial standard of alphitonin (black).

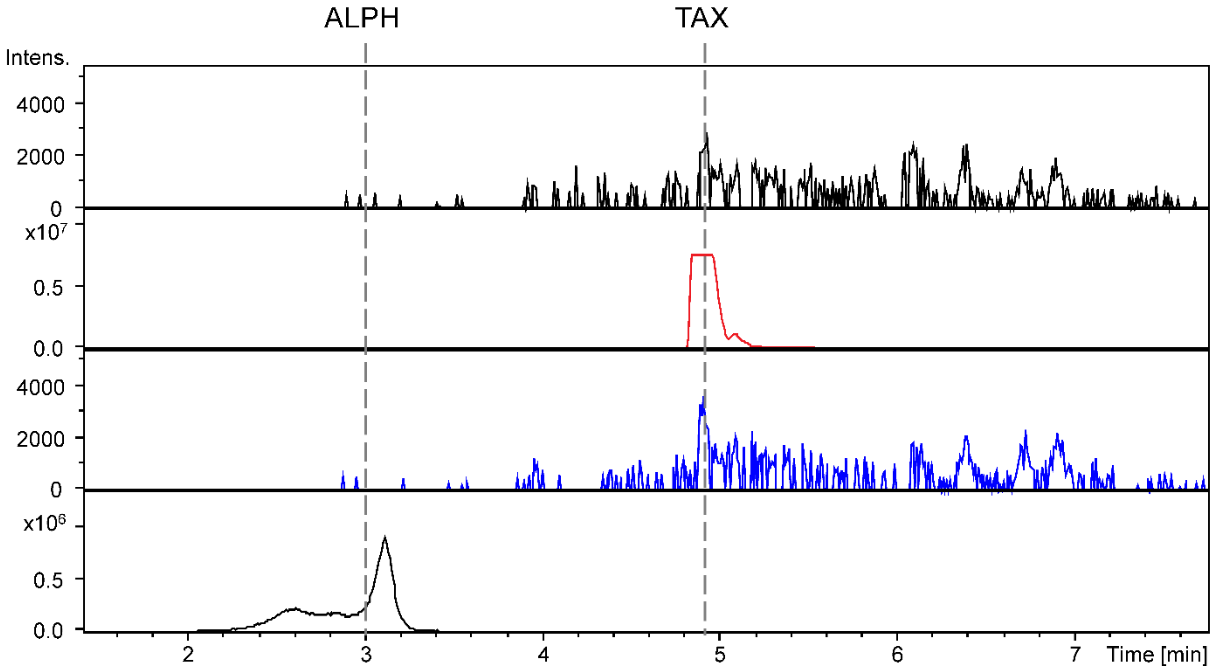

Supplementary Figure 3. Extracted ion chromatograms (EICs) for taxifolin/alphitonin m/z of control samples of *S. albidoflavus* UO-FLAV-004 strain cultures with DMSO (black) or with 100 µM taxifolin feeding (red), and strain UO-FLAV-004-ErCHI with DMSO (dark blue) at time 0 h. Alphitonin commercial standard (black) is shown. ALPH, alphitonin; TAX, taxifolin.

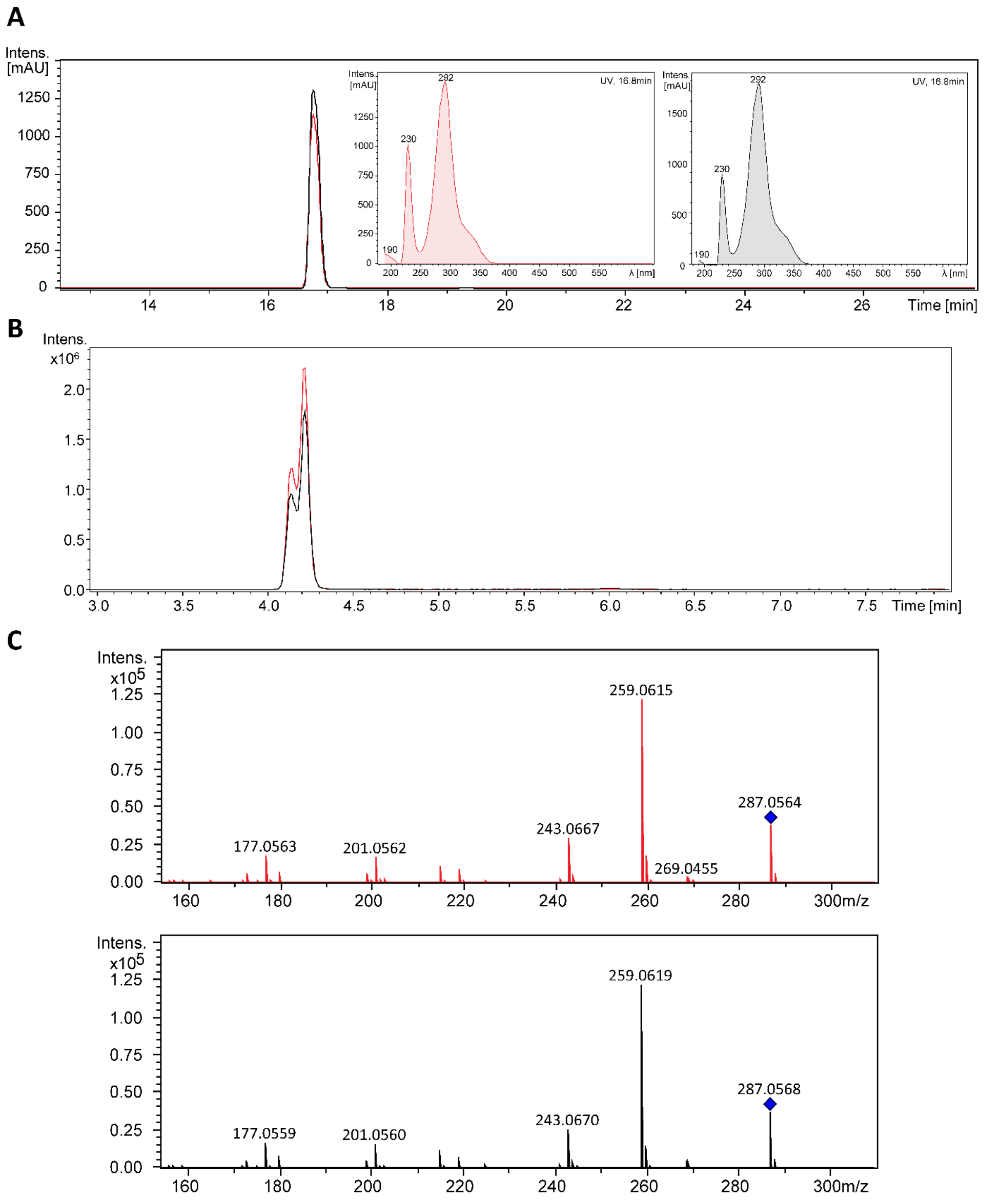

Supplementary Figure 4. Comparisons of pure commercial standards of maesopsin (black) and 2-hydroxynaringenin (red). **(A)** HPLC-DAD chromatograms at 279-281 nm and UV/Vis spectra. **(B)** EICs at m/z 287.0561 ± 0.005 $[M-H]^-$. **(C)** MS/MS spectra.

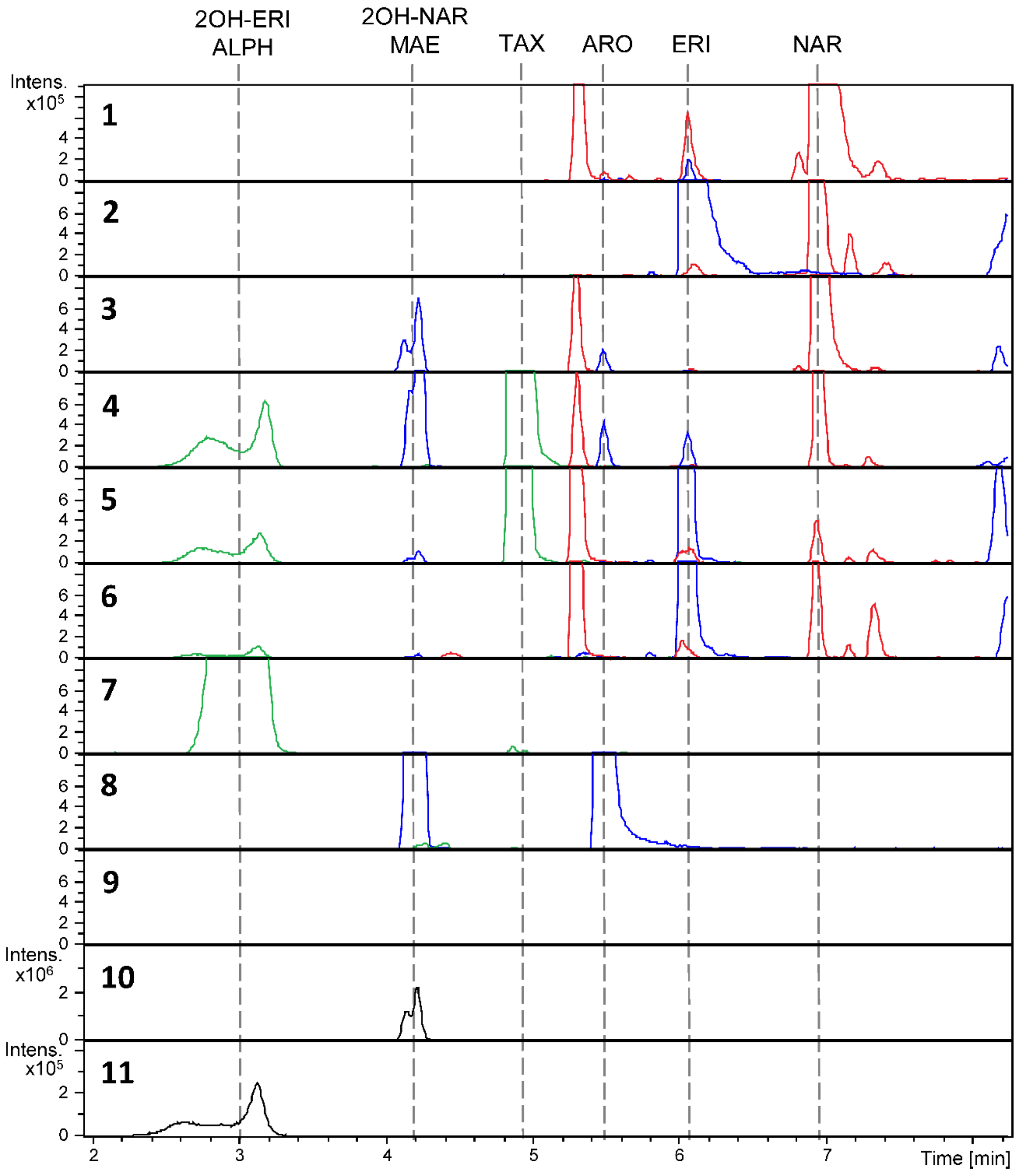

Supplementary Figure 5. EICs for naringenin m/z (red); eriodictyol, aromadendrin, 2OH-NAR and maesopsin m/z (blue); and taxifolin, 2OH-ERI and alphitonin m/z (green) of *S. albidoflavus* strains **(1)** UO-FLAV-004-NAR, **(2)** UO-FLAV-004-ERI, **(3)** UO-FLAV-004-ARO, **(4)** UO-FLAV-004-ARO + 100 µM ERI feeding, **(5)** UO-FLAV-004-TAX, **(6)** UO-FLAV-004-ALPH, **(7)** UO-FLAV-004-ErCHI + 100 µM TAX feeding, **(8)** UO-FLAV-004-ErCHI + 100 µM ARO feeding, **(9)** UO-FLAV-004-ErCHI + DMSO. **(10)** EIC for 2OH-NAR/maesopsin m/z of the corresponding pure commercial standards (black). **(11)** EIC for alphitonin m/z of the pure commercial standard (black). NAR: naringenin; ERI: eriodictyol; ARO: aromadendrin; TAX: taxifolin; 2OH-NAR: 2-hydroxynaringenin; MAE: maesopsin; 2OH-ERI: 2-hydroxyeriodictyol; ALPH: alphitonin.
